# Learning interpretable causal networks from very large datasets, application to 400,000 medical records of breast cancer patients

**DOI:** 10.1016/j.isci.2024.109736

**Published:** 2024-04-16

**Authors:** Marcel da Câmara Ribeiro-Dantas, Honghao Li, Vincent Cabeli, Louise Dupuis, Franck Simon, Liza Hettal, Anne-Sophie Hamy, Hervé Isambert

**Affiliations:** 1 CNRS UMR168, Institut Curie, Université PSL, Sorbonne Université, Paris, France; 2 INSERM U932, Institut Curie, Paris, France; 3 Department of Medical Oncology, Institut Curie, Saint-Cloud, France; 4 Department of Surgery, Institut Curie, Université Paris, Paris, France

**Keywords:** Cancer systems biology, Computing methodology, Health informatics, Machine learning

## Abstract

Discovering causal effects is at the core of scientific investigation but remains challenging when only observational data are available. In practice, causal networks are difficult to learn and interpret, and limited to relatively small datasets. We report a more reliable and scalable causal discovery method (iMIIC), based on a general mutual information supremum principle, which greatly improves the precision of inferred causal relations while distinguishing genuine causes from putative and latent causal effects. We showcase iMIIC on synthetic and real-world healthcare data from 396,179 breast cancer patients from the US Surveillance, Epidemiology, and End Results program. More than 90% of predicted causal effects appear correct, while the remaining unexpected direct and indirect causal effects can be interpreted in terms of diagnostic procedures, therapeutic timing, patient preference or socio-economic disparity. iMIIC’s unique capabilities open up new avenues to discover reliable and interpretable causal networks across a range of research fields.

## Highlights

• We report iMIIC, a reliable, scalable and interpretable causal discovery method
• iMIIC relies on information theory principles to confidently predict causal relations
• iMIIC also distinguishes genuine causes from putative and latent causal effects
• iMIIC performance is shown on synthetic and real-world breast cancer healthcare data

## Graphical abstract

**Figure:**
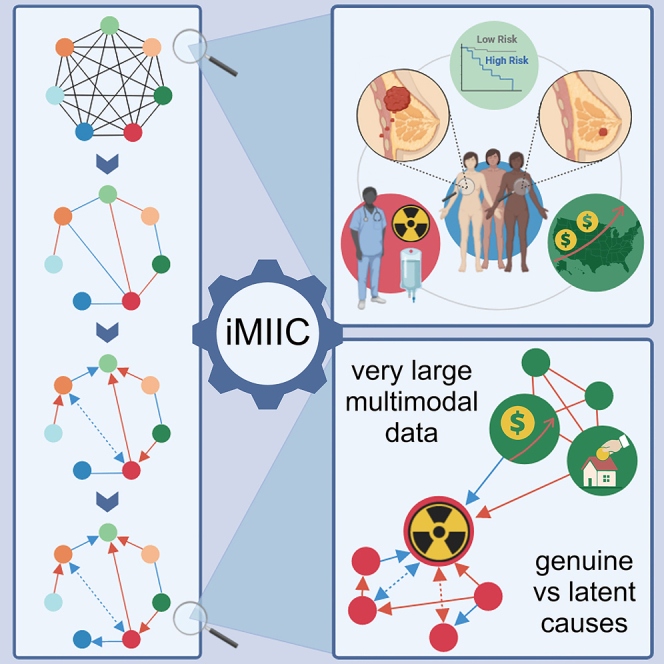

## Introduction

**Figure 1:**
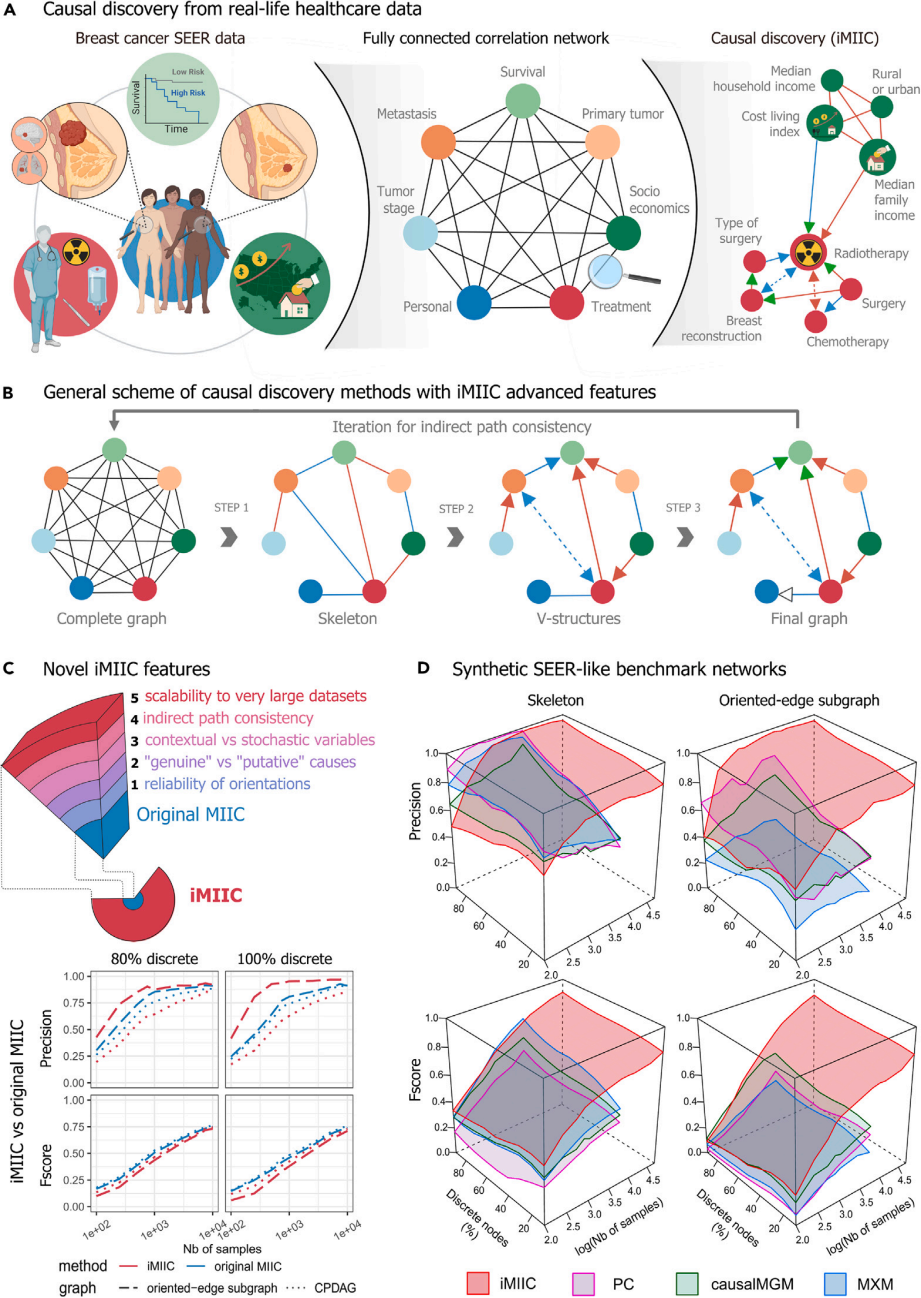
Causal discovery from real-world healthcare data using constraint-based methods (A) SEER database includes 407,791 medical records of breast cancer patients diagnosed between 2010 and 2016. Causal discovery aims at uncovering cause-effect relations across such globally correlated datasets. (B) General scheme of constraint-based methods, including iMIIC’s novel advanced features outlined in Figure S1 workflow and described in method details: Step 1, removal of dispensable edges, while guaranteeing indirect path consistency; Step 2, ‘v-structure’ orientation with reliable orientations and latent common causes shown as bidirected edges; Step 3, propagation of orientation shown with white arrowhead and distinction between ‘putative’ and ‘genuine’ causes, shown with green arrowheads. (C) Novel iMIIC advanced features and benchmark comparison with original MIIC. (D) Synthetic SEER-like benchmark networks with different proportions of discrete variables, see main text, STAR Methods and Figures S5–S7. Created with BioRender.com.

Nationwide medical records contain massive amounts of real-world data on human health, including some personal, familial and socio-economic information, which frequently affect not only health conditions, but also timing of diagnosis, medical treatments and, ultimately, the survival of patients. Besides, such non-medical determinants of human health are usually controlled in clinical trials, which select specific groups of patients through restrictive enrolment criteria. However, the wealth of information contained in real-world medical records remains largely underexploited due to the lack of unsupervised methods and tools to analyze them without preconceived hypotheses. This highlights the need to develop new machine learning strategies to analyze healthcare data, in order to uncover unsuspected associations and possible cause-effect relations between all available information recorded in the medical history of patients, Figure 1A.

Learning cause-effect relations from purely observational data have long been known to be, in principle, possible thanks to seminal works on causal discovery methods.^1^^,^^2^ In essence, causal discovery learns causal graphs by uncovering causal relations from specific correlation patterns involving at least three variables, which goes beyond the popular notion that pairwise correlation does not imply causation. Importantly, causal discovery should be distinguished from causal inference, which aims at quantifying causal effects in terms of hypothetical interventions, assuming that the causal graph is known, but requires additional assumptions (i.e., the identifiability of causal effects), not generally testable in observational studies.^2^ Yet, while observational data account for the vast majority of available datasets across a wide range of domains, causal discovery still remains notoriously challenging in the absence of systematic intervention, which is often impractical, too costly, or unethical when it concerns human health.

**Figure 2:**
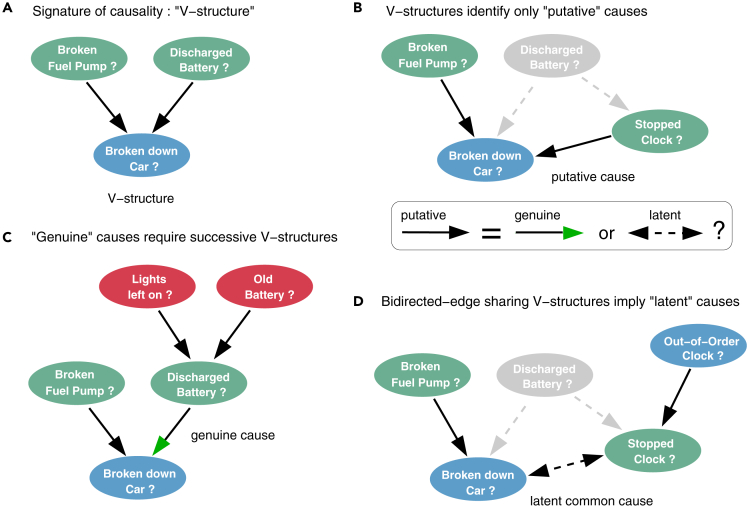
Causal discovery principles from observational data: distinguishing genuine causes from putative and latent causes We outline here the principles to uncover cause-effect relations in a purely observational dataset and distinguish “genuine” causes from “putative” and “latent” causes. The rationale is illustrated on the causally intuitive toy example of an imaginary dataset of old cars. (A) The signature of causality in such observational datasets corresponds to 3-variable “v-structure” subgraphs involving two *independent* and thus *unconnected* possible causes, “Broken fuel pump?” and “Discharged battery?”, and a resulting effect, “Broken down car?”. The converging orientations of this v-structure toward its middle variable, “Broken down car?”, stem from the fact that these two edges cannot be undirected, nor can they point toward either “Broken fuel pump?” or “Discharged battery?”, as these alternative graphical models would imply correlations contradicting the independence between “Broken fuel pump?” and “Discharged battery?”. Alternatively, causal relations can sometimes be uncovered between two variables only, under the specific assumption of continuous additive noise models.^12^ However, in the general case, causal discovery requires at least three and often more variables, as the independence between possible causes in a v-structure is frequently conditional on other variable(s), not considered here, defining a separating set, see method details. Conversely, conditioning on the tip of a v-structure, here “Broken down car?,” induces spurious associations between its independent possible causes.^1^^,^^2^ Likewise, selecting a dataset with specific values for this tip variable results in spurious associations due to selection bias in the dataset,^13^^,^^14^ such as some apparent anti-correlation between different possible causes, “Broken fuel pump?” and “Discharged battery?”, if only “Broken down car? = yes” are selected. (B) However, v-structures remain in fact causally ambiguous^2^ as they only identify “putative” causes, which can either be “genuine” causes, displayed with a green arrowhead, or suggest the presence of unmeasured confounders, i.e., latent common causes unobserved in the dataset and represented with a bidirected edge. For instance, the variable “Clock stopped?,” frequently used as a proxy for “Discharged battery?” also forms a similar v-structure with “Broken fuel pump?”; yet, it is well known that “Clock stopped?” cannot be a genuine cause of “Broken down car?,” as tampering with a car’s clock cannot actually cause a car to break down. (C) In absence of background knowledge and direct intervention on variables, showing that “Discharged battery?” is indeed a genuine cause of “Broken down car?” requires to exclude the possibility of an unobserved common cause (i.e., an unmeasured confounder) between “Discharged battery” and “Broken down car?” To this end, one needs to find another v-structure upstream of “Discharged battery?” (e.g., “Lights left on?” → “Discharged battery?”← “Old battery?”) or to have prior knowledge about an upstream (putative) cause and to show that the effect of at least one upstream variable on the downstream variable “Broken down car?” is entirely *indirect* and mediated (at least in part) by the intermediary variable “Discharged battery?” This requires to find a conditional independence between an upstream variable and “Broken down car?” conditioned on a separating set, which includes the intermediary variable “Discharged battery.” (D) Conversely, ruling out a putative cause as genuine cause requires to show that the relation actually originates from an unobserved common cause by finding a fourth variable (e.g., “Out-of-order clock?”) defining another v-structure, inducing a bidirected edge between “Broken down car?” and “Clock stopped?” with the v-structure in (b). The advanced iMIIC method distinguishes genuine from putative causal edges, as well as, undirected and bidirected edges, by assessing separate head or tail orientation probabilities at each edge extremity (see Figure S1 workflow, results and method details).

While causal discovery is tightly linked to methods designed to learn graphical models,^1^^,^^2^^,^^3^^,^^4^ most structure learning methods are not actually designed to uncover cause-effect relations. In particular, maximum likelihood approaches, such as Search-and-Score^5^ or Graphical Lasso^6^ methods, are restricted to specific model classes, assuming either fully directed graphs or fully undirected graphs, and cannot therefore learn the causal or non-causal nature of graph edges. By contrast, constraint-based causal discovery methods assume broader classes of graphs and can learn the orientation of certain edges solely based on observational data,^1^^,^^2^ Figure 1B. To this end, they first learn structural constraints, in the form of conditional independence relations, which provide indirect and somewhat cryptic information about possible causal relationships between observed as well as unobserved variables, as outlined in Figure 2. Yet, despite being theoretically sound given unlimited amount of data,^7^ constraint-based methods remain unreliable and difficult to interpret on the relatively small datasets, they can handle in practice.

We report here the advanced causal discovery method, iMIIC (interpretable MIIC), that can learn more reliable and interpretable causal graphical models, as well as, handle much larger datasets (e.g., including a few hundred thousand samples). The iMIIC method, outlined in Figure S1 workflow, greatly expands the causal discovery performance of the recent structure learning method, MIIC (Multivariate Information-based Inductive Causation), combining constraint-based and information-theoretic frameworks.^8^^,^^9^^,^^10^ iMIIC’s performance relies on three main conceptual advances and associated methodological developments. First, iMIIC quantitatively improves the reliability of inferred orientations, based on a general information-theoretic principle. It results in only a few percent of false positive orientations on challenging benchmarks adapted from real-world healthcare data. Second, iMIIC is uniquely able to distinguish “genuine” causes from “putative” and “latent” causal effects. This is an essential distinction to disambiguate the causal interpretation of oriented edges in inferred networks, as outlined on an intuitive example in Figure 2. Third, iMIIC quantifies indirect effects, while ensuring their consistency with the global network structure. This is important to interpret indirect contributions in term of indirect paths through the corresponding contributor nodes in the inferred network, which is generally not possible with other causal discovery methods. In addition, iMIIC distinguishes contextual from stochastic variables, which allows the inclusion of externally controlled variables in causal networks, and, finally, iMIIC enables scalability to very large datasets. These unique capabilities open up new avenues to discover reliable and interpretable causal networks across a range of research fields. We demonstrate iMIIC’s causal discovery performance on synthetic and real-world healthcare data originating from more than 400,000 medical records of breast cancer patients from the Surveillance, Epidemiology, and End Results (SEER) program,^11^ Figure S2.

## Results

### Overview and limitations of causal discovery methods

Constraint-based causal discovery methods proceed through successive steps, outlined in Figure 1B. The first step consists in removing, iteratively, all dispensable edges from an initial fully connected network, whenever two variables are independent or conditionally independent given a so-called separating set of conditioning variables. The second step then consists in orienting some of the edges of the undirected graph (named skeleton) to form so-called “v-structures”, X→Z←Y, which are the signature of causality in observational data, Figure 2. Finally, the third step aims at propagating the orientations of v-structures to downstream edges, Figure 1B. However, traditional constraint-based methods lack robustness on finite datasets, as their long series of uncertain decisions lead to an accumulation of errors, which limit the reliability of the final networks. In particular, spurious conditional independences, stemming from coincidental combinations of conditioning variables, lead to many false negative edges and, ultimately, limit the accuracy of inferred orientations. The recent causal discovery method, MIIC,^8^^,^^10^ learns more robust causal graphical models by first collecting iteratively significant information contributors before assessing conditional independences (see method details). In practice, MIIC’s strategy limits spurious conditional independences and greatly improves the sensitivity or recall (i.e., the fraction of correctly recovered edges) compared to traditional constraint-based methods, Figures S3 and S4. In addition, MIIC can handle heterogeneous data (i.e., combining continuous and categorical variables) and missing data,^10^ as well as, unobserved latent variables,^8^ that are ubiquitous in many real-world applications.

Yet, the original MIIC method still presents a number of limitations, that the iMIIC method aims to overcome. In particular, original MIIC (1) presents a lower reliability in predicting edge orientation than edge presence, (2) uncovers “putative” rather than “genuine” causal relations, (3) does not distinguish contextual from stochastic variables, (4) does not guarantee indirect path consistency with the global network structure, and (5) has a limited scalability in the presence of continuous variables. All in all, the iMIIC method, outlined in Figure S1 workflow, is shown to overcome all these limitations, hence greatly enhancing the reliability, interpretability and scalability of causal discovery on large scale synthetic data, as well as, real-world observational datasets.

### iMIIC improves the reliability of inferred orientations

While the original MIIC significantly outperforms traditional constraint-based methods in inferring reliable orientations, a substantial loss in precision usually remains between MIIC skeleton and oriented graph predictions, Figure S4. This is due to orientation errors originating mainly from inconsistent v-structures, X→Z←Y, whose middle node *Z* could also be included in the separating set of the unconnected pair {X,Y}, in contradiction with the head-to-head meeting of the v-structure. Inconsistent v-structures are particularly common for datasets including discrete variables with (too) many levels. To prevent such inconsistent orientations, iMIIC implements more conservative orientation rules, based on a general mutual information supremum principle^15^^,^^16^ regularized for finite datasets, see method details. In practice, it greatly enhances the reliability of predicted orientations with only a small sensitivity loss compared to MIIC original orientation rules, Figure 1C. In particular, iMIIC’s orientation precision exceeds 90% on challenging benchmarks adapted from real-world heterogeneous data, outlined below, when other causal discovery methods typically level off below 50–60% orientation precision at large sample size, Figure 1D (oriented-edge subgraph precision plot) and Figures S5–S7 (dashed lines in precision plots).

### iMIIC distinguishes “genuine” from “putative” causal relations

Traditional constraint-based methods and indeed the original MIIC method merely discover “putative” causal relations, as v-structure orientations are *a priori* compatible with both genuine cause-effect relations and the effects of unobserved common causes, as outlined on an intuitive example in Figure 2. By contrast, iMIIC distinguishes “genuine” from “putative” causal edges by ruling out the effect of an unobserved common cause (or unmeasured confounder) for each predicted genuine causal edge. It is achieved by assessing separate probabilities of arrow head and tail for all oriented edges, see method details and iMIIC’s workflow, Figure S1. Genuine causal edges (represented with a green arrow head) are then predicted if both arrow head and tail probabilities are statistically significant, while causal edges remain “putative” if their tail probability is not statistically significant or cannot be determined from purely observational data. Likewise, bidirected edges, interpreted as the effect of unobserved common causes, correspond to two significant head probabilities, while all other cases are graphically represented as undirected edges.

### iMIIC distinguishes contextual from stochastic variables

The separate probabilistic framework of arrow head versus tail orientations implemented in iMIIC also allows to include prior knowledge about certain head or tail orientations. For instance, including a few contextual variables in graphical models can help specify a control parameter or experimental conditions or characterize the personal profile of patients (e.g., sex, year of birth), depending on the nature of the dataset. Unlike most other variables of the dataset, such contextual variables are not stochastically varying and should have, by assumption, all their edges without incoming arrow head, i.e., $p_{\text{tail}}=1$. This expresses our prior knowledge that contextual variables cannot be the consequence of other observed or unobserved variables in the dataset.

### iMIIC enforces indirect path consistency and quantifies their information contributions

The rationale behind the removal of dispensable edges in the first step of constraint-based causal discovery methods is that all statistical associations between disconnected variables should be graphically interpretable in terms of indirect paths in the final network. However, this is frequently not the case in practice.^17^ In particular, there is no guarantee that the separating sets identified during this iterative removal of edges remain consistent in terms of indirect paths in the final network. To this end, iMIIC adapts a novel algorithmic scheme^17^ to ensure that all separating sets identified to remove dispensable edges are consistent with the final inferred graph. It is achieved by repeating the constraint-based structure learning scheme, iteratively, while selecting only separating sets that are consistent with the skeleton or the partially oriented graph obtained at the previous iteration, as outlined in iMIIC’s workflow, Figure S1. This indirect path consistency improves the interpretability of iMIIC inferred networks in terms of indirect effects, which are also quantified through indirect information contributions, see method details.

### iMIIC outperforms existing methods on synthetic SEER-like benchmark datasets

The performance of iMIIC has been benchmarked against original MIIC as well as other state-of-the-art constraint-based methods on synthetic benchmark datasets resembling the real-world SEER dataset, analyzed below, albeit with varying proportions of discrete versus continuous variables, see SEER-like dataset generation in the quantification and statistical analysis section of Methods. Figure 1C demonstrates that iMIIC significantly improves the precision of orientations to the expense of a relatively small loss in orientation sensitivity and F-score for SEER-like benchmark datasets with large proportions of discrete variables. For instance, for N=500, orientation precision (resp. F-score) already exceeds 85% (resp. 32%) with iMIIC versus 73% (resp. 39%) with original MIIC, for SEER-like benchmark datasets with 80% discrete variables, and even 93% (resp. 25%) versus 64% (resp. 35%) for fully discrete datasets, Figure 1C. In addition, iMIIC greatly outperforms the reliability and sensitivity of inferred orientations against other state-of-the-art constraint-based methods, Figures 1D and S5–S7. In particular, iMIIC’s orientation F-scores are about twice as high as PC algorithm’s^18^^,^^19^ orientation F-scores, for all sample sizes and discrete variable proportions in these SEER-like datasets. For instance, for benchmarks with 80% discrete variables as in the actual SEER dataset, iMIIC already reaches 88% (resp. 44%) in precision (resp. F-score) for $N=10^{3}$, against about 60% (18%) for conservative PC,^19^^,^^20^ 50% (36%) for causalMGM^21^ and 24% (18%) for MXM.^22^ For $N=10^{4}$, iMIIC reaches 92% (73%) in precision (F-score), against about 75% (40%) for conservative PC, 62% (55%) for causalMGM and 30% (30%) for MXM. Finally, iMIIC reaches more than 90% for both orientation precision and F-score, for $N=10^{5}$, which is beyond the sample size attainable by other methods. See Benchmark results in the quantification and statistical analysis section of Methods for comparisons with higher proportion of continuous variables.

### Application to nationwide breast cancer medical records

We applied iMIIC on a large breast cancer dataset^11^ from the SEER program of the National Cancer Institute, which collects data on cancer diagnoses, treatment and survival for ∼ 35% of the US population, Figure 1A. Breast cancer^23^ is the most common invasive cancer in women and is curable in only 70–80% of patients with large disparities in terms of tumor subtypes and stages at diagnostic, initial and subsequent treatments, as well as patient’s age, ethnicity, genetic predisposition, lifestyle or socio-economic situation. Numerous retrospective association studies^24^^,^^25^^,^^26^^,^^27^ and a few causal inference investigations^28^^,^^29^^,^^30^^,^^31^ have been reported on SEER’s cancer data, making it a unique benchmark resource to assess the actual performance of causal discovery methods on real-world healthcare data.

**Figure 3:**
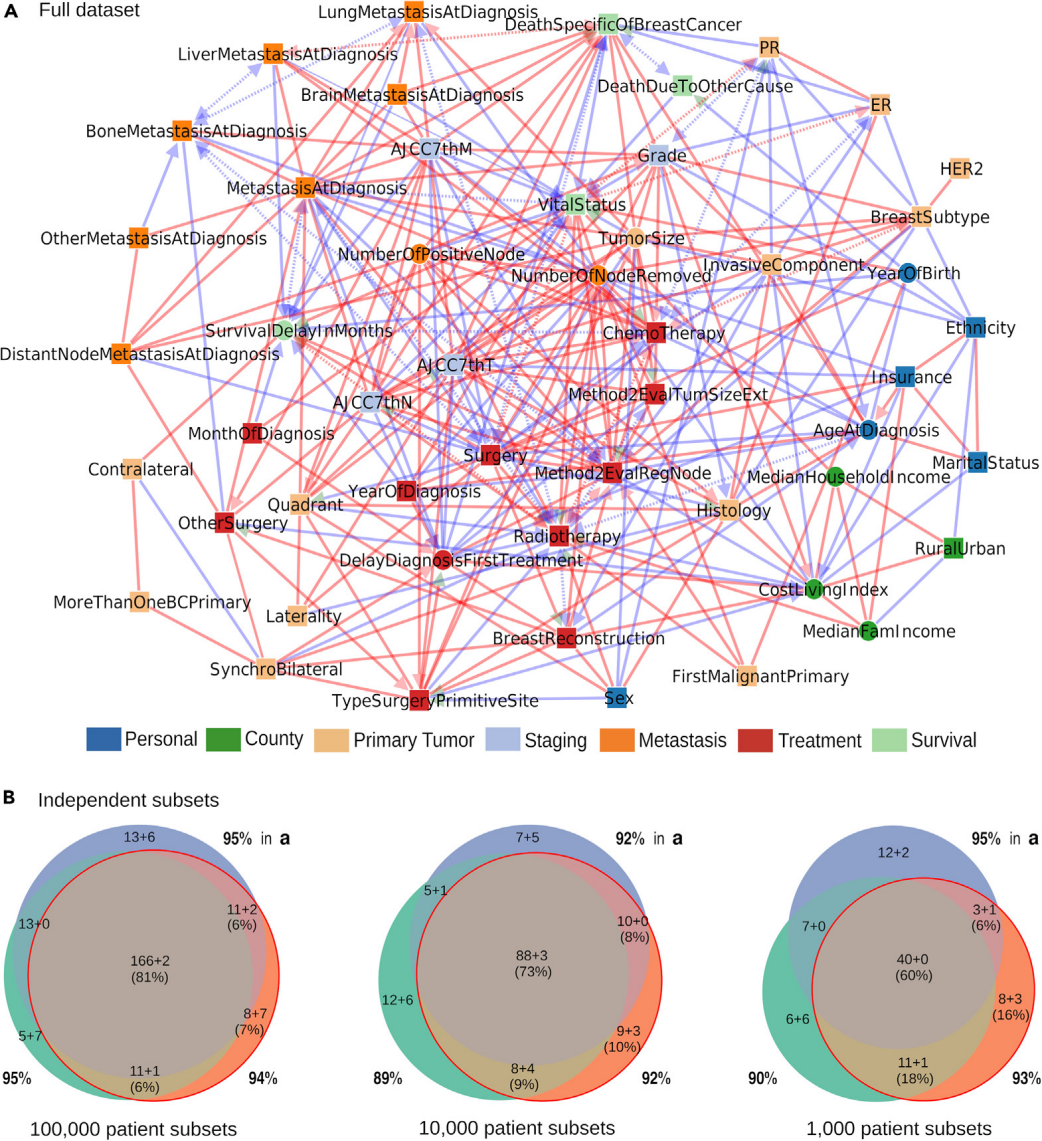
SEER breast cancer networks inferred by iMIIC (A) The 51-node network inferred by iMIIC from SEER dataset including 396,179 breast cancer patients diagnosed between 2010 and 2016. This skeleton consistent network contains 280 edges and includes 2 contextual variables, Sex and Year of birth. The corresponding orientation consistent network contains 340 edges, Figure S8. Red (resp. blue) edges indicate correlated (resp. anticorrelated) variables. ‘Genuine’ causal edges are shown with green arrowheads and ‘putative’ causal relations with red or blue arrowheads, while bidirected dashed edges correspond to the effect of unobserved latent variables (Figure 2). See Table S1 for a list and causal nature of each edges predicted by iMIIC. (B) Comparisons of networks inferred from three independent sub-samplings of the same size of 100,000, 10,000 or 1,000 patient subsets (from left to right). Number of shared edges (regardless of orientations) in the Euler diagrams are given as a sum a+b where *a* (resp. *b*) corresponds to the number of edges included in (resp. absent from) the full dataset network in (a). Percentages in brackets refer to the subset network with the median total number of edges (red circle). The fractions of edges also found in the full network in (a) are indicated around the Euler diagram for each independent subset. These fractions of shared edges are high (i.e., 89–95%) for all independent subset sizes, demonstrating that iMIIC reconstructs essentially subnetworks of the full network for all independent subset sizes, with a decreasing overlap between smaller subnetworks learnt from smaller independent subsets, see main text.

We present here iMIIC’s causal discovery analysis on SEER breast cancer data for the period 2010–2016. There are 407,791 medical records but only 396,179 distinct patients due to multiple breast primary tumors for some patients. Fifty-one clinical, socio-economic and outcome variables have been selected for their relevance to breast cancer and for their limited redundancy or missing information, Figure S2. The resulting breast cancer network, Figure 3A, provides an interpretable graphical model including 280 edges, for which most cause-effect relations are either known or can be ruled out based on common or expert knowledge as well as clinical practice. This expert knowledge validation is further supported by an independent statistical validation based on different sub-samplings of the 396,179-patient dataset, Figure 3B. We present these complementary validations in the next section before addressing the causal interpretation of iMIIC breast cancer network in the following sections.

### Independent validations of iMIIC breast cancer network

The expert knowledge validation of the causal effects inferred by iMIIC is summarized in Table S1 and indicates that about 90% of predicted genuine or putative causal effects are correct, while an additional 8% of cause-effect relations seem plausible, based on clinical and epidemiological knowledge. Hence, iMIIC’s novel orientation rules lead to only 2% of erroneous causal edges, as compared to about 15% when MIIC’s original orientation rules are applied to analyze the same ∼ 400,000 patient SEER cohort. This expert knowledge validation of iMIIC results mainly relies on independent background information not included in the SEER dataset, such as the typical chronology of care pathways of patients and the results of a range of randomized clinical trials (e.g., to assess the effect of chemotherapy on patient survival). Besides, none of the predicted genuine causal edges connect pairs of non-cancer-specific variables, such as personal or socio-economic information, that are susceptible to a possible selection bias^13^^,^^14^ through breast cancer diagnosis (Figure 2). In addition, unmeasured (latent) confounders can be ruled out for genuine causal edges (Figure 2) while contributions by measured confounders are estimated as indirect path contributions (see method details). Yet, other sources of bias in data collection and analysis have been reported on the SEER database^32^^,^^33^ as discussed in the following section.

Independently from this expert knowledge validation, we also assessed the statistical robustness of iMIIC breast cancer network by performing a range of independent sub-samplings from 100,000 patient subsets down to 1,000 patient subsets, Figure 3B. It showed that 89–95% of the edges of each smaller network, learned from these 4- to 400-fold smaller subsets of patients, are in fact included in the full dataset network, Figure 3A. Hence, smaller networks learned from independent subsets of the full dataset are essentially subnetworks of the full network with a gradually decreasing proportion of shared edges between smaller networks learned from smaller independent subsets, Figure 3B. This interesting finding demonstrates the multiplicity of equally possible subnetworks for smaller independent subsets of the full dataset, while validating statistically the robustness of iMIIC inferred networks for very large datasets. In particular, the full network Figure 3A includes 90% of all the combined edges of three networks learned from three independent subsets of 100,000 patients, Figure 3B. In addition, 88% of the edge orientation probabilities are compatible between the three 100,000-patient subset networks and 92% of those are also compatible with the edge orientation probabilities of the full network, Table S1. Similar statistically robust results are found for iMIIC orientation consistent network, Figure S8 and Table S1.

### Causal interpretation of iMIIC breast cancer network

We now address the clinical and socio-economic interpretation of the SEER breast cancer network inferred by iMIIC, Figure 3A. We will focus, in particular, on the expected as well as more surprising genuine causal relations uncovered by iMIIC, and will propose interpretations of the counter-intuitive cause-effect predictions in terms of care pathway, therapeutic decisions, patient preferences or socio-economic determinants of healthcare. We present these results from the perspective of different classes of variables and associated subnetworks, starting with the survival subnetwork, then the primary tumor subnetwork, the surgery and subsequent treatment subnetwork, and finally the socio-economic subnetwork.

#### Survival subnetwork

**Figure 4:**
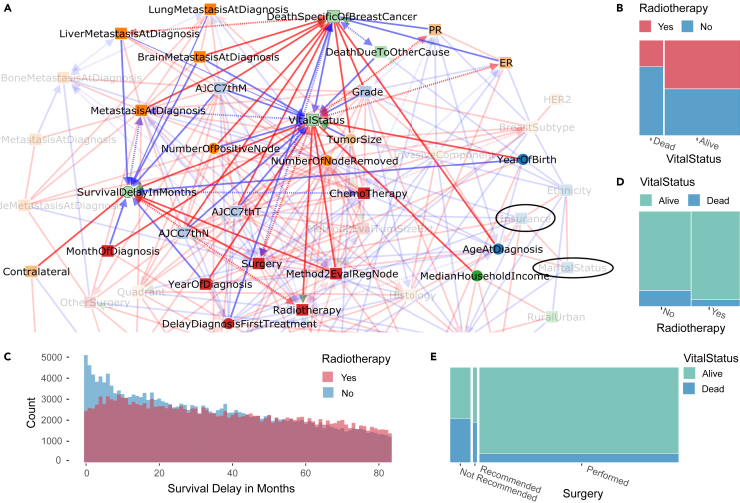
Survival subnetwork inferred by iMIIC from SEER breast cancer dataset (A) Subnetwork highlighting direct relations with survival variables (VitalStatus, DeathSpecificOfBreastCancer, DeathDueToOtherCause, SurvivalDelayInMonths). The absence of direct links with other variables (such as Insurance and Marital Status highlighted in the network) can be interpreted in terms of indirect path contributions consistent with the network skeleton, see main text and method details. (B) Joint distribution of Radiotherapy and Vital Status highlighting the counter-intuitive causal relation between them, see text. (C) Histogram of Survival Delay In Months for patients having received Radiotherapy or not. Each bin represents one month. The early blue peak suggests that a number of patients died within 3–6 months after diagnosis, hence, before they could receive Radiotherapy, in agreement with the causal direction predicted in (a). This results in an over-estimated apparent benefit of Radiotherapy in (d), see main text. (D) Joint distribution of Vital Status and Radiotherapy. (E) Joint distribution of Vital Status and Surgery.

**Figure 5:**
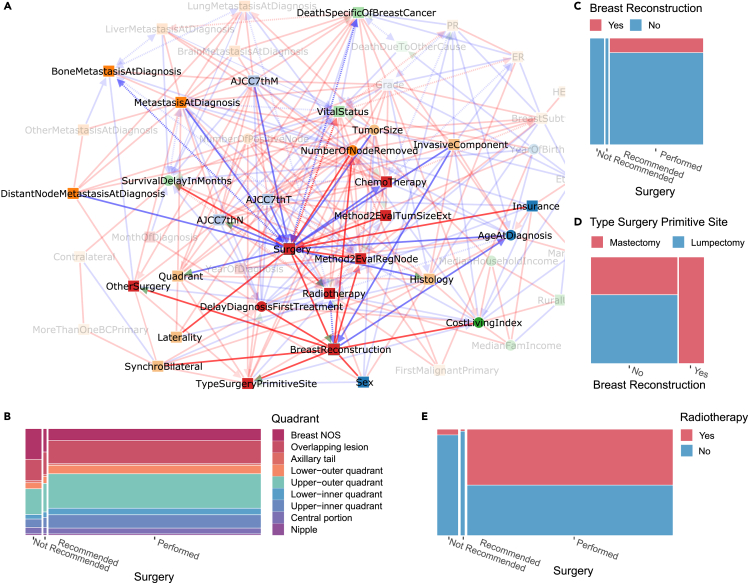
Surgery and subsequent treatments subnetwork inferred by iMIIC from SEER breast cancer dataset (A) Subnetwork highlighting direct relations with Surgery and Breast Reconstruction. (B) Joint distribution of Quadrant and Surgery. (C) Joint distribution of Breast Reconstruction and Surgery. (D) Joint distribution of Type Surgery Primitive Site and Breast Reconstruction. (E) Joint distribution of Radiotherapy and Surgery. See main text for causal interpretation of the role of Surgery on refining primary tumor characterization and subsequent therapeutic decisions including personal choice of patients.

The full network, Figure 3A, contains four nodes associated with patient survival status at the end of 2016 and defining a survival subnetwork, that includes all variables directly linked to patient survival status, Figure 4A. Beyond the vital status of each patient (dead or alive), two additional nodes specify the cause of death, either from breast cancer or from any other cause, and a third continuous variable corresponds to the survival or follow-up delay in months, subjected to the censoring period 2010–2016 of the study. Figure 4A shows that known factors responsible for the death due to breast cancer are correctly recovered by iMIIC, such as metastasis at diagnosis (overall mortality rate 49.2%), with the worse distant metastases at diagnosis (brain and liver) also retaining direct links to both Death specific to breast cancer and Vital status, which accounts for their excess mortality rates, i.e., brain metastasis (70.5%) and liver metastasis (59.5%). Similarly, the number of metastasis-positive lymph nodes and the staging variables (AJCC7th T, N, and M) are all correctly connected to both death specific to breast cancer and vital status, and not to any other cause of death. By contrast, iMIIC infers causal relations between year of birth and death due to other cause, as well as, year of birth and vital status, as expected. We can also note that the deaths of patients, irrespective of their cause, are rightly predicted to lead to a reduction in their survival delays. Yet, Figure 4A contains also less intuitive findings. In particular, vital status is robustly inferred to ‘cause’ radiotherapy, both in the full dataset and in all three 100,000 patient subsets, with 51% of alive patients having undergone radiotherapy against only 27% of dead patients, Figure 4B. This suggests that early death within the first few months after diagnosis may prevent radiotherapy for some patients who might have otherwise received this treatment, have they lived longer. This short term causal effect between vital status and radiotherapy is consistent with the rapid decline of the survival delay distribution for the first 3–6 months in absence of radiotherapy, Figure 4C, which corresponds to the typical range of delays for radiotherapy after diagnosis, depending on whether it is performed as second treatment after surgery or as third treatment after both surgery and chemotherapy.^34^ All in all, this short term causal effect of vital status on radiotherapy outweighs the causally reversed, beneficial effect of radiotherapy on the long term survival of patients. This suggests a strong “immortal time bias”^32^ in the apparent benefit of radiotherapy, Figure 4D, which would need to be corrected with the “landmark method”^32^^,^^35^ excluding patients dying within a specified period after surgery, or by emulating a target trial from observational data.^36^ By contrast, surgery –which is typically performed within 5–8 weeks after diagnosis– is found to be the primary cause leading to the prolonged survival delay of patients, as discussed below, Figures 4E and 5A.

Finally, we note that a number of variables that have been reported to be associated to survival variables are in fact indirectly rather than directly connected to them. This is, in particular, the case of insurance^37^^,^^38^ and marital status.^39^^,^^40^ The indirect contributions between Death due to breast cancer and all the other recorded variables of the dataset have been quantitatively estimated using Equation 10 in method details. The results are listed in Table S2. In particular, the indirect effect of Insurance (with uninsured/Medicaid/non-Medicaid as categories) on Death due to breast cancer is shown to be indirectly explained through Surgery (50%), Chemotherapy (14%), Marital status (20%), Radiotherapy (9%), and Breast reconstruction (7%). Similarly, the indirect effect of marital status (with single/married/separated/divorced/widowed categories) on Death due to breast cancer is shown to be indirectly explained through Surgery (57%), Year of birth (40%), and Ethnicity (3%). In fact, Surgery accounts for the main indirect contributions between Death due to breast cancer and a number of other variables, such as synchro-bilateral tumors (Surgery [67%]), tumor with an invasive component (Surgery [73%]), tumor histology (Surgery [73%]) and Breast reconstruction (Surgery [90%]), Table S2. These quantitative indirect contributions highlight the importance of Surgery as a significant mediator or covariate of breast cancer survival.

Beyond the specific outcome variable, Death due to breast cancer, the indirect contributions between all pairs of variables have also been quantified by iMIIC. The exhaustive list can be found on the online result page of the full network at https://miic.curie.fr/job_results_showcase_2022.php?id=SEER2022 (See Summary tab, which lists the indirect contributions, as well as other computed information, on all 1,069 pairs of non-independent variables).

#### Primary tumor subnetwork

Besides metastasis at diagnosis, the Estrogen and Progesterone Receptor (ER/PR) status and the size of the primary tumor are also found to directly affect the vital prognosis of patients, Figure S9A. In particular, iMIIC infers that ER status reduces the risk of death due to breast cancer from 17.7% (ER-) to 5.4% (ER+), with a large indirect contribution (82%) from PR status. This is consistent with the ER transcriptional control of PR^41^ and a significantly higher mortality rate of ER+/PR- patients (11.8%) than ER+/PR+ patients (4.4%). Likewise, PR status is also the main indirect contribution between Breast cancer subtype and Death due to breast cancer (PR [86%]) and between Chemotherapy and Death due to breast cancer (PR [68%]), Table S2. This highlights the overlooked importance of PR status relative to ER status on breast cancer prognosis for specific cancer subtypes, such as luminal A and B, and associated treatments. Indeed, breast cancer subtypes are classified, using a nonspecific hormone receptor status HR and the HER2 receptor status, as luminal A (HR+/HER2-), luminal B (HR+/HER2+), HER2 enriched (HR-/HER2+), and Triple negative (HR-/HER2-), where HR-stands for both ER- and PR-, while HR+ stands for either ER+ or PR+, as if ER and PR status could mutually back up each other. However, iMIIC results specifically highlight and quantify the direct as well as indirect contributions of PR status to patient survival.

In addition, iMIIC infers a number of direct associations between the histology of primary tumors and other variables, Figure S9A, such as Age at diagnosis (in agreement with early reports^42^) and with synchro bilateral primaries (detected within 6 months of first diagnosis) which are almost twice more likely to occur when lobular carcinoma is present, Figure S9B. By contrast, no significant association is found with contralateral primary tumors detected more than 6 months after diagnosis, Figure S9C.

#### Surgery and subsequent treatment subnetwork

Interestingly, iMIIC also uncovers the central role of Surgery on the precise characterization of primary tumors, Figure 5A. For instance, iMIIC uncovers a somewhat unexpected genuine causal link from Surgery to Histology, which reflects that histological types are frequently refined after surgery by the pathologist based on the surgical specimen. This is consistent with a significant increase in histological types including specific tissues after surgery such as Infiltrating duct mixed with other types of carcinoma (+77% after surgery), Infiltrating duct and lobular carcinoma (+48%), Infiltrating duct carcinoma, NOS (+7.6%), and a corresponding decrease in more generic histological types such as Lobular carcinoma, NOS (−11%), Carcinoma, NOS (−91%), and Adenocarcinoma, NOS (−95%), where NOS stands for Not Otherwise Specified. Similarly, iMIIC rightly infers that the staging variable, AJCC7thN, is usually based on the pathological report following surgery, while not performing surgery (due to the presence of distant metastases at diagnosis or the patient’s old age) leads to much more frequent unspecified breast quadrant localization for primary tumor, Figure 5A, i.e., 30.4% “Breast NOS” when surgery is not recommended versus 11.1% when it is performed, Figure 5B.

Likewise, iMIIC uncovers the central role of Surgery on the therapeutic decisions about subsequent treatments, such as breast reconstruction and radiotherapy, Figure 5A. While breast reconstruction indeed requires breast surgery, Figure 5C, iMIIC also correctly infers that the Type of Surgery at the Primary Site (lumpectomy or mastectomy) largely depends on the personal choice of early stage breast cancer patients between breast conservation and reconstruction alternatives, Figures 5A–5D. Similarly, iMIIC rightly infers that radiotherapy is a frequent “consequence” of breast surgery, Figure 5A, i.e., 53% versus 4% radiotherapy if surgery is performed or not, Figure 5E, especially after lumpectomy (75%) to limit the risk of relapse after breast conservation surgery.

#### Socio-economic subnetwork

**Figure 6:**
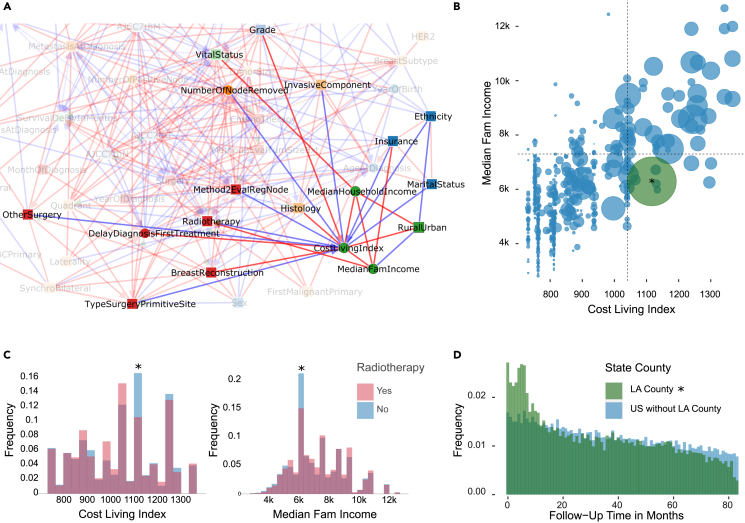
Socio-economic subnetwork inferred by iMIIC from SEER breast cancer dataset (A) Subnetwork highlighting direct relations with socio-economic county variables (CostLivingIndex, MedianFamIncome, MedianHouseholdIncome, and RuralUrban). (B) Bubble plot of the joint distribution of Median Family Income and Cost of Living Index. The bubble area represents the number of patients in that county. Dashed lines correspond to the mean Cost of Living Index and mean Median Family Income. The green bubble with an asterisk corresponds to Los Angeles (L.A.) county which accounts for 10% of the full dataset. (C) Histograms of Cost of Living Index and Median Family Income grouped by Radiotherapy. Bins with an asterisk correspond to L.A. county. (D) Histograms of Follow-Up Time in Months for L.A. patients and for all other US counties included in SEER.

The full breast cancer network on Figure 3A includes four socio-economic variables pertaining to the county of residence of each patient: Median Family Income, Median Household Income, Cost of Living Index and the Rural-Urban population size of each county. These four socio-economic variables actually form a fully connected subgraph (i.e., a clique), indicating their strong interdependencies, and are directly connected to a number of other variables, Figure 6A. Interestingly, Vital Status is only connected to this county variable clique through Median Household Income, which is consistent with earlier reports on the association between life expectancy and incomes.^43^ By contrast, all other patient specific variables connected to the county clique (such as tumor grade, radiotherapy, breast reconstruction, insurance) have in fact at least one link with Cost of Living Index, highlighting the healthcare system integration into the global economy. In particular, there is a direct association between higher cost of living and more favorable breast cancer prognosis (e.g., fewer invasive components at diagnosis). This is presumably due to better preventive healthcare including easier access to breast cancer screening centers and more comprehensive insurance coverage. Yet, there are also strong disparities between counties, as manifested by the opposite associations of Insurance and Radiotherapy with Median Family Income versus Cost of Living Index, Figure 6A. These intriguing findings can be traced back to Los Angeles (L.A.) county, amounting to about 10% of the whole dataset, which presents a lower than average median family income (29–38% percentile range) despite a higher than average cost of living index (58–67% percentile range), Figure 6B. This must have led to an exacerbated financial burden for many of the 39,089 breast cancer patients diagnosed in L.A. county between 2010 and 2016. Although 18% of these patients benefited from Medicaid insurance (as compared to 10% in the whole dataset), many had to opt for affordable but limited private insurance including significant co-payment policies or even to become uninsured especially before the application of the Affordable Care Act in January 2014 (3.4% uninsured in 2013 against 1.5% in 2014). As a result, many L.A. patients appear to have renounced to undergo expensive treatments. In particular, only 32.6% of patients underwent radiotherapy in L.A. as compared to 50% of patients nationwide excluding L.A. county, Figure 6C, which can only be partly accounted for by county differences in under-reported radiotherapy of outpatients.^32^^,^^33^ Moreover, an estimated 7% of L.A. patients even appear to have dropped out of therapy or moved to a different county not included in SEER database (against 1.5% nationwide, excluding L.A. county), based on the rapidly decreasing follow-up time distribution in L.A. as compared to the rest of the dataset, Figure 6D. This corresponds to the fraction of patients having had their last medical contact less than a year after diagnosis and more than a year before the end of this study in December 2016.

## Discussion

Nationwide healthcare data, such as the SEER breast cancer dataset analyzed here, are especially interesting from a methodological point of view; they provide real-world benchmark datasets, which can help assess the reliability of causal discovery methods on real-world data, as most cause-effect predictions can be validated or dismissed, based on expert knowledge, clinical practice or possible data collection and selection biases. Besides, the interpretability of Machine Learning methods is particularly relevant for applications on clinical data, for which Artificial Intelligence assisted recommendations can hardly rely on black box classifiers only and need to be explainable in terms of intelligible rationales to medical practitioners. Yet, beyond clinical data, causal discovery methods have the potential to become essential Machine learning approaches to interpret diverse observational data in a wide range of domains, for which systematic perturbation experiments are not available due to practical, cost or ethical reasons. In particular, causal discovery can guide biological research by predicting the causal effects of specific interventions,^44^^,^^45^^,^^46^^,^^47^ such as gene expression or gene silencing, which can then be probed by targeted siRNA, gene knock-out or CRISPR-based editing experiments.

In the context of SEER’s breast cancer dataset, we presented iMIIC results in the form of four subnetworks (Figures 4, 5, 6, and S8) focusing on specific classes of variables (such as survival, primary tumor, treatment and socio-economic variables) and their direct interactions in the global network, Figure 3A. This is primarily due to practical reasons given the limited number of possible figures. However, iMIIC detailed predictions have been thoroughly investigated through both expert assessment and statistical validation. In particular, all genuine and putative causal edges have been assessed and essentially all validated through expert knowledge, Table S1. In addition, we also performed an exhaustive quantification of indirect contributions between all pairs of (connected or unconnected) variables of the global 51-node network, such as between insurance, marital status, ER vs*.* PR status and survival to breast cancer, Table S2. Assessing all causal edges as well as indirect information contributions between all pairs of variables provides a unique and comprehensive interpretation of SEER breast cancer dataset and inferred network, which goes beyond the analysis of a few variables of interest usually performed in large biomedical studies (e.g., patient survival versus treatment and a few covariates).

Hence, iMIIC provides a detailed and validated interpretation across all variables selected in this nation-wide cohort of nearly 400,000 breast cancer patients. This exhaustive analysis uncovers many expected causal relations, such as the adverse consequence of metastasis and the protecting effect of ER+ and specifically PR+ status on death due to breast cancer, or the fact that year of birth is the primary reason for death due to other causes by the end of the study. On the other hand, the effects of insurance coverage or marital status, which have been reported to reduce the risk of death due to breast cancer, are found to be entirely indirect and mainly mediated by treatments (60–80%), notably, surgery (> 50%). In fact, surgery appears as the cornerstone of breast cancer therapy by first helping refine histological types, then guide therapeutic decisions on radiotherapy and breast reconstruction and ultimately prolong the survival delays of patients. Yet, iMIIC also correctly infers that the type of surgery (lumpectomy or mastectomy) at the primary site largely depends on the personal choice of early stage breast cancer patients between breast conservation or reconstruction alternatives. By contrast, other treatments, such as radiotherapy and chemotherapy, seem to have less decisive impacts on breast cancer outcome, which might be due in part to some under-reported treatment information in the SEER database.^32^^,^^33^ Radiotherapy even appears to be a consequence, not a cause, of vital status, suggesting that early death within the first few months after diagnosis may prevent radiotherapy for some patients who might have otherwise received this treatment, have they lived longer. Finally, iMIIC recovers direct associations between socio-economic county variables (such as median family income and cost of living index) and patient specific variables (such as tumor grade, radiotherapy, breast reconstruction, insurance), highlighting the healthcare system integration into the global economy. While higher costs of living are on average associated to more favorable cancer prognosis, presumably due to better preventive healthcare and more comprehensive insurance coverage, iMIIC also uncovers large disparities between family income and cost of living indices across counties (e.g., for L.A. county), leading to exacerbated financial burden with patients giving up expensive treatments or even dropping out of treatment.

In summary, iMIIC is a general causal discovery method, which uncovers direct and possibly causal relations as well as network consistent indirect effects for a broad range of biological and clinical data. Importantly, iMIIC is fully unsupervised and does not need preconceived hypothesis nor expert knowledge. In particular, iMIIC automatically adjusts for measured confounders (in the form of indirect contributions) and distinguishes genuine causes from putative and latent causal effects by either ruling out or highlighting the effect of unmeasured confounders for each causal edge (Figures 2 and S1). While iMIIC is not immune to possible data collection and selection biases, which can affect observational data, it is based on a robust information theoretic framework, making it particularly reliable to interpret challenging types of data, such as heterogeneous data including combination of continuous and categorical variables integrated from different sources (e.g., clinical, personal, socio-economic data, as demonstrated here and on much smaller datasets in earlier studies^10^^,^^48^) or different experimental techniques (e.g., single cell transcriptomics^8^^,^^44^^,^^45^^,^^46^ and imaging data^10^^,^^47^). In principle, iMIIC could be applied to a wide range of other domains to uncover causal relations and quantify indirect contributions when only observational data is available. With the advent of virtually unlimited datasets in many data science domains, scalable causal discovery methods are much needed and we believe that iMIIC can bring unique insights based on causal interpretation across a range of research fields.

### Limitations of the study

This study comes with limitations. First, iMIIC is a causal discovery method, not a causal inference method. More specifically, iMIIC can discover genuine causes, solely based on observational data and their multivariate information, and distinguish genuine causes from putative and latent causal effects. However, iMIIC does not perform causal inference, which aims at quantifying causal effects in terms of hypothetical interventions assuming that the causal graph is known, but requires additional assumptions (i.e., identifiability of causal effects), not generally testable in observational studies.^2^ In particular, the causal effects of a putative cause are nonidentifiable, implying that the results of intervention on a putative cause cannot be quantified from observational data alone. Yet, instead of quantifying causal effects, iMIIC’s information-theoretic framework quantifies information contributions from indirect paths, while ensuring their consistency with the global network structure. This provides quantitative estimates of indirect information contributions in all settings without the requirement of identifiability. Another limitation of iMIIC, shared by all causal discovery methods, is that not all causal effects can be discovered from purely observational data due to Markov equivalence between alternative causal networks. This highlights the importance of achieving the highest possible precision with iMIIC in order to limit the number of false positives amongst the subset of causal relations that can actually be uncovered from purely observational data. Finally, iMIIC results and interpretations might also be affected by data collection and selection biases, as discussed in the results section, although this was largely ruled out in the present study.

## STAR★Methods

### Key resources table

**Table:** 

REAGENT or RESOURCE	SOURCE	IDENTIFIER
**Software and algorithms**		

R	https://www.r-project.org	
iMIIC online server	https://miic.curie.fr	
iMIIC R package	https://github.com/miicTeam/miic_R_package	
pcalg	https://r-forge.r-project.org/projects/pcalg	
bnlearn	https://cran.r-project.org/web/packages/bnlearn	
rCausalMGM	https://github.com/tyler-lovelace1/rCausalMGM	
MXM	https://cran.r-project.org/web/packages/MXM	

### Resource availability

#### Lead contact

Further information and requests for resources should be directed to and will be fulfilled by the lead contact, Hervé Isambert (herve.isambert@curie.fr).

#### Materials availability

This study did not generate new unique reagents.

#### Data and code availability

• The dataset of breast cancer patients was obtained from the Surveillance, Epidemiology and End Results program, which can be accessed at https://seer.cancer.gov/seertrack/data/request/. The script implementing preprocessing steps is provided as Data S1. The benchmark data generation codes and synthetic SEER-like datasets are provided as Data S2.
• Causal discovery using iMIIC was performed on the open access server https://miic.curie.fr, with highlighted novel iMIIC features, or running the R package available at https://github.com/miicTeam/miic_R_package. Other R packages used for benchmark comparisons are available at the URLs listed in the key resources table.

### Method details

#### Preprocessing of SEER breast cancer data

There are 407,791 breast cancer records in SEER for the period 2010-2016, but only 396,179 distinct patients due to multiple breast primary tumors for some patients. For each patient, we selected the first breast primary tumor recorded in SEER and indicated the total number of breast cancer primaries during the 2010-2016 period in the variable *MoreThanOneBCPrimary*. *SynchroBilateral* was also engineered to identify patients who had tumors in both breasts diagnosed within less than 180 days of each other, while *Contralateral* identifies patients who had a subsequent tumor in the other breast diagnosed more than 180 days after the first breast tumor primary. Some categorical variables had some of their categories merged, either because these categories had the same general meaning or because they were too rare amongst patients (*i.e.* < 0.1% of patients excluding those with missing data for the considered variable). These variables include *Ethnicity, TypeSurgeryPrimitiveSite, Surgery, OtherSurgery, OtherMetastasisAtDiagnosis, Insurance and Histology*. Hence, categories recorded in less than 0.1% of patients were merged and renamed to ‘Other’. *BreastReconstruction* was engineered based on *TypeSurgeryPrimitiveSite* (*i.e.* SEER surgery code ranges 43-49, 53-59, 63-69, and 73-75 were assigned ‘Yes’, while other surgery codes were assigned ‘No’). *Radiotherapy* was created from *Radiation sequence with surgery*, that has much less missing data (0.05%) than the original *Radiation* variable (49%). *TumorSize* merges two distinct variables that contained tumor sizes for years 2004-2015 and 2016+, respectively. Likewise, the largely missing 2016 information for the *MetastasisAtDiagnosis* variable was recovered based on information contained in specific metastasis variables (*i.e. BoneMetastasisAtDiagnosis, LungMetastasisAtDiagnosis, LiverMetastasisAtDiagnosis, BrainMetastasisAtDiagnosis, OtherMetastasisAtDiagnosis*). Finally, *MedianFamIncome* and *MedianHouseHoldIncome* are the average of these continuous variables over the periods 2007-2011, 2008-2012, 2009-2013, 2010-2014, 2011-2015, and 2012-2016. The script implementing these preprocessing steps is provided as Data S1.

#### Overview and limitations of constraint-based methods

Constraint-based methods^1^^,^^2^ proceed through successive steps, outlined in Figure 1B, whose accuracy ultimately conditions the reliability and interpretability of the final causal graphical model. Starting from a fully connected graph, their first step consists in removing, iteratively, all dispensable edges whenever two variables are marginally independent or conditionally independent given a so-called separating set of conditioning variables. Positive (resp. negative) partial correlations are represented with red (resp. blue) edges in Figure 1B and all other network figures. The rationale behind this first step is that all statistical associations between disconnected variables in the predicted graph should be graphically interpretable in terms of indirect paths through their separating set. This is, however, frequently not the case in practice.^17^

The second step then consists in orienting some of the edges of the undirected graph (named skeleton) obtained at the first step, based on the signature of causality in observational data. This amounts to orient so-called “v-structures” as, X→Z←Y, whenever the edge X−Y has been removed without including a common neighbor *Z* of *X* and *Y* in their separating set, S. The converging orientations of such a v-structure graphically indicate that *Z* cannot be a cause of neither *X* nor *Y*, which would otherwise require *Z* to be included in the separating set, S. However, this does not imply that *X* (or *Y*) is an actual cause of *Z*, which also requires to rule out the possibility that the direct link between *X* and *Z* (or *Y* and *Z*) might in fact originate from an unmeasured confounder, that is, from a latent common cause, *L*, unobserved in the dataset, *i.e.* X←L→Z, as described with an intuitive example in Figure 2. Finally, the third step aims at propagating the orientations of v-structures to downstream edges, to fulfill the assumptions of the underlying graphical model class of constraint-based methods.

However, while traditional constraint-based methods have been shown to be theoretically sound and complete given an unlimited amount of data,^7^ they lack robustness on finite datasets, as their long series of uncertain decisions lead to an accumulation of errors, which limit the reliability of the final networks. In particular, spurious conditional independences, stemming from coincidental combinations of conditioning variables, lead to many false negative edges and, ultimately, limit the accuracy of inferred orientations.

#### Overview and limitations of MIIC method

The recent causal discovery method, MIIC, combines constraint-based and information-theoretic frameworks to learn more robust causal graphical models.^8^^,^^10^ To limit the accumulation of errors in removing dispensable edges, MIIC does not directly attempt to uncover conditional independences but, instead, iteratively subtracts the most significant information contributions of successive contributors, $A_{1}$, $A_{2}$, …, $A_{n}$, from the mutual information between each pair of variables, I(X;Y), as,

(Equation 1) $$I\left(X;Y\right)-I\left(X;Y;A_{1}\right)-I\left(X;Y;A_{2}|A_{1}\right)-\cdots -I\left(X;Y;A_{n}|{\left\{A_{i}\right\}}_{n-1}\right)=I\left(X;Y|{\left\{A_{i}\right\}}_{n}\right)$$

where $I\left(X;Y;A_{k}|{\left\{A_{i}\right\}}_{k-1}\right)>0$ is the *positive* information contribution from $A_{k}$ to I(X;Y), that is not dependent on the first k−1 collected variables, ${\left\{A_{i}\right\}}_{k-1}$.^49^^,^^50^ Conditional independence is eventually established when the residual conditional mutual information on the right hand side of Equation 1, $I\left(X;Y|{\left\{A_{i}\right\}}_{n}\right)$, becomes smaller than a complexity term, *i.e.* $k_{X;Y|\left\{A_{i}\right\}}\left(N\right)⩾I\left(X;Y|{\left\{A_{i}\right\}}_{n}\right)⩾0$, which depends on the considered variables and sample size *N*. This complexity term also defines size corrected (or “regularized”) conditional mutual information as,

(Equation 2) $$I^{\prime }\left(X;Y|{\left\{A_{i}\right\}}_{n}\right)=I\left(X;Y|{\left\{A_{i}\right\}}_{n}\right)-k_{X;Y|\left\{A_{i}\right\}}\left(N\right)$$

which become *negative* under conditional independence (*i.e.* $I^{\prime }\left(X;Y|{\left\{A_{i}\right\}}_{n}\right)⩽0$), that is, whenever sufficient and significant indirect positive contributions could be iteratively collected in Equation 1 to warrant the removal of edge XY.

This leads to an undirected skeleton, which MIIC then (partially) orients based on the sign and amplitude of the regularized conditional 3-point information terms,^8^^,^^49^ corresponding to the difference between regularized conditional mutual information terms.

(Equation 3) $$I^{\prime }\left(X;Y;Z|\left\{A_{i}\right\}\right)=I^{\prime }\left(X;Y|\left\{A_{i}\right\}\right)-I^{\prime }\left(X;Y|\left\{A_{i}\right\},Z\right)$$

In particular, negative conditional 3-point information terms, $I^{\prime }\left(X;Y;Z|\left\{A_{i}\right\}\right)<0$, correspond to the signature of causality in observational data^49^ and lead to the prediction of a v-structure, X→Z←Y, if *X* and *Y* are not connected in the skeleton (with $I^{\prime }\left(X;Y|\left\{A_{i}\right\}\right)⩽0$). By contrast, a positive conditional 3-point information term, $I^{\prime }\left(X;Y;Z|\left\{A_{i}\right\}\right)>0$, implies the absence of a v-structure and suggests to propagate the orientation of a previously directed edge X→Z-Y as X→Z→Y (with $I^{\prime }\left(X;Y|\left\{A_{i}\right\},Z\right)⩽0$), to fulfill the assumptions of the underlying graphical model class.

In practice, MIIC’s strategy to circumvent spurious conditional independences significantly improves the sensitivity or recall, that is, the fraction of correctly recovered edges, compared to traditional constraint-based methods, Figure S4. However, original MIIC as well as all other causal discovery methods still present a number of major limitations, such as (*i*) a lower reliability in predicting edge orientation than edge presence, (*ii*) a poor scalability, notably with continuous or mixed-type data, (*iii*) a remaining ambiguity on the “putative” *versus* “genuine” causal nature of oriented edges (Figure 2), and (*iv*) a frequent inconsistency of separating sets with respect to indirect paths in the inferred network. The advanced iMIIC method, outlined in Figure S1 workflow, overcomes all these limitations, as detailed in the following sections.

#### Improved reliability of iMIIC inferred orientations

While the original MIIC significantly outperforms traditional constraint-based methods in inferring reliable orientations, a substantial loss in precision usually remains between MIIC skeleton and oriented graph predictions, Figure S4. This is due to orientation errors originating from inconsistent v-structures, X→Z←Y, whose middle node *Z* could also be included in the separating set of the unconnected pair {X,Y}, in contradiction with the head-to-head meeting of the v-structure. In particular, for discrete variables with (too) many levels, complexity terms can easily outweigh (conditional) mutual information for weakly dependent variables. As a result, original MIIC tends to infer some v-structure orientations, X→Z←Y, for which both (conditional) mutual information terms in Equation 3 are negative, *i.e.* $I^{\prime }\left(X;Y|\left\{A_{i}\right\}\right)<I^{\prime }\left(X;Y|\left\{A_{i}\right\},Z\right)<0$, suggesting that *Z* could in fact be included in a separating set of the {X,Y} pair, in contradiction with the inferred v-structure, X→Z←Y. To circumvent this issue, iMIIC implements more conservative orientation rules by essentially treating categorical and continuous variables alike, based on a general mutual information supremum principle,^15^^,^^16^ outlined below. In particular, Theorem 1, below, requires to rectify all negative regularized (conditional) mutual information, defining (conditional) independence (*e.g.* $I^{\prime }\left(X;Y|\left\{A_{i}\right\}\right)⩽I^{\prime }\left(X;Y|\left\{A_{i}\right\},Z\right)⩽0$), to null values (*i.e.* $I^{\prime }\left(X;Y|\left\{A_{i}\right\}\right)=I^{\prime }\left(X;Y|\left\{A_{i}\right\},Z\right)=0$), which leads to vanishing conditional 2-point and 3-point information in Equation 3, for the example above, and prevents the orientation of all inconsistent v-structures.

##### General mutual information supremum principle

Estimating (conditional) mutual information between continuous or mixed-type variables is notoriously more challenging than between categorical variables.^51^^,^^52^ Original MIIC computes regularized mutual information between continuous or mixed-type variables through an optimum discretization scheme, based on a general mutual information supremum principle^15^ regularized for finite datasets and using an efficient $O\left(N^{2}\right)$ dynamic programming algorithm.^10^ This approach finds optimum partitions, P and Q, specifying the number and positions of cut-points of each continuous variable, *X* and *Y*, to maximize the regularized mutual information between them,

(Equation 4) $$I^{\prime }\left(X;Y\right)=\underset{P,Q}{\sup}I^{\prime }\left({\left[X\right]}_{P};{\left[Y\right]}_{Q}\right)$$

Such optimization-based estimates of mutual information are at par with alternative distance-based k-nearest neighbor (kNN) approaches^51^^,^^52^ but have also the unique advantage of providing an effective independence test to identify independent continuous or mixed-type variables.^10^ This is achieved when partitioning *X* and *Y* into single bins maximizes the regularized mutual information in Equation 4, which vanishes exactly in this case, *i.e.* $I^{\prime }\left(X;Y\right)=I^{\prime }\left({\left[X\right]}_{1};{\left[Y\right]}_{1}\right)=0$ if $I^{\prime }\left({\left[X\right]}_{P};{\left[Y\right]}_{Q}\right)⩽0$ for all partitions P,Q. By contrast, kNN estimates still need an actual independence test to decide whether some variables are effectively independent or not, as kNN mutual information estimates are never exactly null.

Yet, the optimum partitioning principle (Equation 4) only applies to mutual information,^15^ *not* conditional mutual information, which need to be estimated through the *difference* between optimum regularized mutual information terms, as $I^{\prime }\left(X;Y|U\right)=I^{\prime }\left(Y;\left\{X,U\right\}\right)-I^{\prime }\left(Y;U\right)=I^{\prime }\left(X;\left\{Y,U\right\}\right)-I^{\prime }\left(X;U\right)$.^10^ As a result of numerical approximation, the regularized conditional mutual information estimates between conditionally independent variables can sometime be negative and lead to inconsistent v-structure orientations, as discussed for discrete data above.

The general mutual information supremum principle,^15^ regularized for finite datasets in Equation 4, is theoretically valid for any type of variable, not just continuous variables. In particular, it could be applied to datasets including discrete or categorical variables with (too) many levels. This would result in the merging of rare levels to better estimate mutual information and conditional mutual information between weakly dependent discrete variables. Ultimately, mutual information estimates between independent discrete variables should lead to the merging of each variable into a single bin, thereby, resulting in regularized mutual information estimates to vanish exactly in this case, as observed for continuous variables. As a result, optimum regularized mutual information should be non-negative as well as, by extension, regularized conditional mutual information, as proved below.

***Theorem 1***. Regularized (conditional) mutual information derived from the general mutual information supremum principle are non-negative.

***Proof***. We first address optimum regularized mutual information without conditioning variables, noting that $I^{\prime }\left(X;Y\right)⩾I^{\prime }\left({\left[X\right]}_{1};{\left[Y\right]}_{1}\right)=0$, where ${\left[X\right]}_{1}$ and ${\left[Y\right]}_{1}$ are the *X* and *Y* variables partitioned into single bins, which leads to a vanishing regularized mutual information, as both mutual information and complexity cost are null for single bin partitions.^49^

Then, regularized conditional mutual information is defined as the *difference* between optimum regularized mutual information terms as, $I^{\prime }\left(X;Y|U\right)=I^{\prime }\left(Y;\left\{X,U\right\}\right)-I^{\prime }\left(Y;U\right)=I^{\prime }\left(X;\left\{Y,U\right\}\right)-I^{\prime }\left(X;U\right)$. However, partitioning *X* and *Y* into single bins leads to $I^{\prime }\left(Y;\left\{X,U\right\}\right)⩾I^{\prime }\left(Y;\left\{{\left[X\right]}_{1},U\right\}\right)=I^{\prime }\left(Y;U\right)$ and $I^{\prime }\left(X;\left\{Y,U\right\}\right)⩾I^{\prime }\left(X;\left\{{\left[Y\right]}_{1},U\right\}\right)=I^{\prime }\left(X;U\right)$ thus implying $I^{\prime }\left(X;Y|U\right)⩾0$.

Hence, Theorem 1 requires to rectify all negative values of regularized (conditional) mutual information, indicating (conditional) independence, to null values instead. Enforcing this rectification of regularized (conditional) mutual information terms is found to significantly enhance the reliability of iMIIC predicted orientations, in particular for datasets with high proportions of discrete variables, with only a small sensitivity loss compared to MIIC original orientation rules, Figure 1C.

#### Improved scalability of iMIIC computations

The computational scalability of iMIIC compared to classical constraint-based methods first relies on the 3off2 strategy^49^^,^^50^ implemented in original MIIC, which iteratively takes off the most significant information contributions from successive contributors, Equation 1, until a robust conditional independence is possibly found, as outlined in Figure S1 workflow. Computationally, this is inherently more efficient than the combinatorial search for conditional independence, performed by classical constraint-based methods, which is also prone to spurious conditional independence, as emphasized above. Yet, despite its improved scalability compared to classical constraint-based methods, original MIIC still presents computational complexity and stability issues with very large datasets, which the novel iMIIC algorithm effectively overcomes.

First, the running time of original MIIC scales linearly with sample size for discrete datasets^8^ but at best quadratically with sample size for continuous or mixed-type datasets,^10^ due to a $O\left(N^{2}\right)$ dynamic programming optimization of the number and positions of cut points to estimate (conditional) multivariate information. This quadratic scaling becomes prohibitive for very large datasets, such as the SEER dataset analyzed here. To circumvent this scalability issue, iMIIC enforces a maximum number of 50 bins, so that the overall optimization of multivariate information estimates remains close to linear in terms of sample size, see Figures S5–S7. While introducing a maximum number of bins slightly underestimates mutual information between strongly associated variables in very large datasets (*i.e.* when the actual supremum is achieved with more bins in Equation 4), it has in fact very little effect on iMIIC’s results in practice. In particular, the critical first step assessing (conditional) independence between variables in step 1a and step 1b of iMIIC’s workflow, Figure S1, is achieved by partitioning these variables into single bins, as discussed above, and is therefore completely insensitive to the actual maximum number of bins in the algorithm.

A second scalability issue concerns the estimation of orientation probabilities by the original MIIC, which are numerically too close to be reliably compared for very large datasets and require to introduce scalable orientation scores and novel definitions of induced tail and head orientation scores, as detailed now.

##### V-structure orientation scores

Head orientation probabilities of v-structures, X∗→Z←∗Y, are computed from negative regularized (conditional) 3-point information, $I^{\prime }\left(X;Y;Z|\left\{A_{i}\right\}\right)<0$, as,^8^

(Equation 5) $$P\left(x\;*\to \underline{z}\right)=P\left(\underline{z}\leftarrow *\;y\right)=\frac{1+e^{N\;I^{\prime }\left(X;Y;Z|\left\{A_{i}\right\}\right)}}{1+3e^{N\;I^{\prime }\left(X;Y;Z|\left\{A_{i}\right\}\right)}}⩾\frac{1}{2}$$

where the end mark (∗) stands either for a head (>), a tail (−) or is undefined (∘), and $e^{N\;I^{\prime }\left(X;Y;Z|\left\{A_{i}\right\}\right)}$ corresponds to the probability ratio between a non-v-structure and a v-structure, $e^{N\;I^{\prime }\left(X;Y;Z|\left\{A_{i}\right\}\right)}=P_{\to -}/P_{\to \leftarrow }=P_{-\leftarrow }/P_{\to \leftarrow }=P_{--}/P_{\to \leftarrow }$. However, due to numerical precision Equation 5 cannot rank orientation probabilities that are too close to 1 for large *N* and iMIIC resorts instead to equivalent v-structure orientation scores,

$${\text{score}}_{v}=-NI^{\prime }\left(X;Y;Z|\left\{A_{i}\right\}\right)+\log\;1\;p\left(e^{N\;I^{\prime }\left(X;Y;Z|\left\{A_{i}\right\}\right)}\right)-\log\;2$$

(Equation 6) $$P\left(x\;*\to \underline{z}\right)=P\left(\underline{z}\leftarrow *\;y\right)=\frac{1}{1+e^{-{\text{score}}_{v}}}$$

which enable the ordering of orientation probabilities, $P_{1}$ and $P_{2}$ between alternative v-structures ($v_{1}$ and $v_{2}$), even for very large *N*, as $0⩽{\text{score}}_{1}<{\text{score}}_{2}<\infty$ is equivalent to $0.5⩽P_{1}<P_{2}<1$.

##### Induced tail and head orientation scores

Similarly, induced orientation probabilities originating from an existing arrowhead $\underline{z}\leftarrow *\;y$ can be estimated through the following probability decomposition formula,^8^

(Equation 7) $$P\left(x*-•\underline{z}\right)=P\left(x*-•\underline{z}|\underline{z}\leftarrow *y\right)P\left(\underline{z}\leftarrow *y\right)+P\left(x*-•\underline{z}|\underline{z}-*y\right)P\left(\underline{z}-*y\right)$$

where • stands for a tail [resp. a head] depending on the positivity [resp. negativity] of $I^{\prime }\left(X;Y;Z|\left\{A_{i}\right\}\right)$ and a corresponding (conditional) independence $I^{\prime }\left(X;Y|\left\{A_{i}\right\},Z\right)⩽0$ [resp. $I^{\prime }\left(X;Y|\left\{A_{i}\right\}\right)⩽0$].

However, using the full probability decomposition above can lead to a higher confidence in tail or head induced probabilities than in the head probabilities they derive from, due to the Markov equivalence of non-v-structures. In addition, induced tail / head probabilities become numerically difficult to compare for large *N*, as Equation 7 cannot be expressed in the form of Equation 6. To circumvent these issues and capture the rationale that the confidence in induced tail / head orientations can only be lower than the confidence in the arrowhead from which they derive, iMIIC redefines the induced tail / head probabilities by retaining only the first term in the probability decomposition above, that is, by assuming that the arrowhead $\underline{z}\leftarrow *\;y$ exists,

(Equation 8) $$P\left(x*-•\underline{z}\right)=P\left(x*-•\underline{z}|\underline{z}\leftarrow *y\right)P\left(\underline{z}\leftarrow *y\right)=\frac{1}{1+e^{-N\left|I^{\prime }\left(X;Y;Z|\left\{A_{i}\right\}\right)\right|}}\times \frac{1}{1+e^{-score_{v}}}=\frac{1}{1+e^{-score_{i}}}$$

where we introduced a rectified induced ${\text{score}}_{i}$,

(Equation 9) $${\text{score}}_{i}=\max\left(0,m-\log\;1\;p\left(e^{-M+m}+e^{-M}\right)\right)$$

$$m=\min\left(N\left|I^{\prime }\left(X;Y;Z|\left\{A_{i}\right\}\right)\right|,{\text{score}}_{v}\right)$$

$$M=\max\left(N\left|I^{\prime }\left(X;Y;Z|\left\{A_{i}\right\}\right)\right|,{\text{score}}_{v}\right)$$

to enable a global numerical ranking of v-structure orientation and induced orientation probabilities even for very large *N* with $0.5⩽P_{1}<P_{2}<1$ corresponding to $0⩽{\text{score}}_{1}<{\text{score}}_{2}<\infty$.

In addition, when orientation propagation is enforced (*i.e.* step 2&3 in iMIIC’s workflow, Figure S1), an induced tail probability can also be “propagated”, as a head probability, to the other end of the edge, if its end mark is still undefined, *i.e.*, $P\left(\underline{x}\leftarrow z\right)=P\left(x\circ -\underline{z}\right)$. However, this orientation propagation rule does not rely on specific information in the available data but rather aims at fulfilling the structural assumptions of benchmark graphical models. Hence, propagation has been applied in benchmark comparisons (Figures 1C, 1D, and S4–S7) but discarded to analyze real-world data (Figures 3, 4, 5, 6, S8, and S9), in order to ensure that causal discovery on real-world applications is solely based on information actually contained in the available data.

#### Orientation confidence and causal nature of edges

Having fully ordered orientation probabilities, even for very large *N*, enables to implement edge orientations in decreasing order of confidence rather than any arbitrary order, as implemented in traditional constraint-based methods. In addition, iMIIC allows also to use an orientation confidence threshold 1>β⩾0.5 to enhance the precision of predicted head and tail orientations and, thereby, our confidence in the causal nature of oriented edges. Hence, a genuine causal relation (represented with a green arrow-head) is predicted if the edge can be assigned both significant head and tail probabilities, $P_{h}>\beta$ and $P_{t}>\beta$, while a putative causal relation is inferred if only one significant head probability can be assessed given the available observational data, *i.e.* $P_{h}>\beta$ and $P_{t}⩽\beta$, as outlined in iMIIC’s workflow for β=0.5, Figure S1. Similarly, a bidirected edge, suggesting the effect of an unobserved common cause, is predicted for two significant head probabilities, while all other cases are graphically represented as undirected edges. In practice, orientation precision threshold β mostly impacts the orientations derived from small datasets and has little effects on large datasets such as SEER presented here. All causal discovery benchmark results have also been obtained without enhancing orientation precision (*i.e.* using β=0.5) which yields a better balance between precision and recall for all sample sizes. Finally, iMIIC also allows to include prior knowledge about certain head or tail orientations in graphical models, for instance, to specify contextual variables (*e.g.* sex, year of birth), which cannot be the consequence of other observed or unobserved variables in the dataset, as outlined in the main text.

#### Indirect path consistency and information contribution

As mentioned in the overview and limitations, above, traditional constraint-based methods, as well as, the original MIIC method do not control for the global structural consistency of their inferred networks. In particular, there is no guarantee that the separating sets identified during the iterative removal of edges (step 1 in Figure 1B) remain consistent in terms of indirect paths in the final network. To this end, iMIIC adapts a novel algorithmic scheme^17^ to ensure that all separating sets identified to remove dispensable edges are consistent with the final inferred graph. It is achieved by repeating the constraint-based structure learning scheme, iteratively, while searching for separating sets that are consistent with the graph obtained at the previous iteration, as outlined in iMIIC’s workflow, Figure S1, until a limit circle of networks is obtained and the union of these graphs is taken as final consistent network.^17^ We define two levels of indirect path consistency: skeleton *versus* orientation consistencies. Skeleton consistency guarantees that any node in a separating set is on an indirect path between the extremities of the corresponding removed edge (regardless of orientations along the path), while orientation consistency further enforces that each node in a separating set is a non-descendent neighbor of at least one of these extremities. Importantly, implementing skeleton or orientation consistency of separating sets can be done at a limited complexity cost, through the use of block-cut tree decomposition of graphs.^17^ All in all, iMIIC indirect path consistency improves the interpretability of the inferred network in terms of indirect effects, which are also quantified with indirect information contributions, based on Equation 1 including finite size corrections from Equation 2,

(Equation 10) $$\text{IndC}\left(A_{k};XY|{\left\{A_{i}\right\}}_{k-1}\right)=\frac{I^{\prime }\left(X;Y;A_{k}|{\left\{A_{i}\right\}}_{k-1}\right)}{I^{\prime }\left(X;Y\right)}$$

with $\sum_{k}^{n}\text{IndC}\left(A_{k};XY|{\left\{A_{i}\right\}}_{k-1}\right)=100\%-I^{\prime }\left(X;Y|{\left\{A_{i}\right\}}_{n}\right)/I^{\prime }\left(X;Y\right)$, where $I^{\prime }\left(X;Y|{\left\{A_{i}\right\}}_{n}\right)/I^{\prime }\left(X;Y\right)$ is the residual fraction of mutual information (*i.e.* not accounted for by $A_{1},A_{2},\cdots ,A_{n}$ indirect contributions given by Equation 10), which vanishes if the XY edge has been removed, that is, if $I^{\prime }\left(X;Y|{\left\{A_{i}\right\}}_{n}\right)=0$, after negative value rectification.

### Quantification and statistical analysis

#### SEER-like dataset generation

SEER-like synthetic datasets were generated using network structures inferred from 10,000 patient subsets of the full SEER dataset of breast cancer patients, to allow for comparison with other causal discovery methods, as detailed below. Random network skeletons of similar SEER-like degree distributions with additional ±2 connection variability at each node were first obtained using a Monte Carlo graph generation algorithm.^53^ These skeletons were subsequently oriented to obtain Directed Acyclic Graphs using a random ordering of their nodes and assigning various proportions of discrete *versus* continuous variables. The marginal distributions of variables without parents were chosen to resemble typical SEER-like marginal distributions, Figure S2, and the other variables were simulated using mixed-type structural equation models (SEMs),^10^ see *e.g.* Figure S3. For each discrete node proportion (decile steps), 25 benchmark networks were obtained and used to generate 100,000 samples each. Benchmark data generation codes and synthetic SEER-like datasets are provided as Data S2.

#### Causal discovery scores

For evaluation purposes, network reconstruction was treated as a binary classification task and classical performance measures, Precision (Prec=TP/(TP+FP)), Recall (Rec=TP/(TP+FN)) and F-score (F=2×Prec×Rec/(Prec+Rec)), were computed to evaluate (*i*) skeleton, (*ii*) completed partially directed acyclic graph (CPDAG) and (*iii*) oriented-edge subgraph reconstructions. CPDAG scores use the same metrics as skeleton scores but rating as “false positive” the erroneous orientation of non-oriented edges in the CPDAG and the non-orientation or opposite orientation of oriented edges in the CPDAG. However, these errors are not equivalent from a causal discovery perspective. Hence, we introduced oriented-edge subgraph scores, that are restricted to the subgraphs containing only oriented edges in the theoretical CPDAG *versus* the inferred graph. These oriented-edge scores, highlighted in the benchmark comparisons (Figures 1C, 1D, and S4–S7), are designed to specifically assess the method performance on causal discovery, that is, on the (genuine and putative) causal edges which can in principle be learnt from observational data *versus* those effectively predicted by the causal structure learning method. Note, however, that the oriented-edge subgraph scores do not distinguish between genuine and putative causes, since none of the causal discovery methods benchmarked against iMIIC can distinguish genuine from putative causes.

#### Benchmarked causal discovery methods

Five causal discovery methods able to analyze mixed-type datasets have been compared over SEER-like generated datasets:

• *Interpretable MIIC (iMIIC)* was run with default parameters for all settings.
• *Original MIIC*^9^^,^^10^ was run with default parameters for all settings (Figures 1C and S4).
• *PC*^18^ from the *pcalg* package^19^ was run with the stable option^54^ and either majority rule^54^ (Figure S4) or conservative rule^20^ (Figures 1D and S5) for orientations. The “ci.test” function from the *bnlearn* package^55^ was used as independence test for mixed-type data (with either “mi-cg” option for discrete against continuous variables, “mi” for discrete against discrete variables or “mi-g” for continuous against continuous variables) and the threshold for significance testing was set to the default α=0.01.
• *causalMGM*^21^ was run with the *rCausalMGM* R package. The initial graph was computed using the mgm() function with each of the 3 lambda parameters equal to 0.05 and the orientations were then obtained with the pcMax() function with default α=0.01 parameter (Figure S6).
• *MXM*,^22^ a mixed-PC constraint-based method, was run using the *MXM* R package. The graph was obtained using the pc.skel() function for skeleton with the “comb.mm” independence test and the default α=0.01 threshold for significance testing and with the pc.or() function for orientations (Figure S7).

#### Computation time

Benchmarks were stopped when the average computation time of a method reached 1 hour per network with high proportion of continuous variables (resp. about 10 minutes per network with low proportion of continuous variables), corresponding to a maximum running time of about 115h for the 250 generated networks at each sample size.

#### Benchmark results

The performance of iMIIC has been benchmarked against state-of-the-art constraint-based methods: PC, MXM and causalMGM, on SEER-like benchmark datasets with different proportions of discrete variables, Figures 1D and S5–S7. Results for datasets with 80% discrete variables, corresponding to the actual proportion in the real-world SEER breast cancer dataset, are discussed in the main text. Similarly, for larger proportions of continuous variables, Figures 1D and S5–S7 demonstrate that iMIIC greatly outperforms the reliability and sensitivity of predicted orientations against state-of-the-art constraint-based methods. For instance, for SEER-like benchmark datasets with only 20% of discrete variables, iMIIC already reaches 81% (resp. 64%) in precision (resp. F-score), for $N=10^{3}$, against 53% (29%) for conservative PC, 50% (40%) for causalMGM and 29% (25%) for MXM. For $N=10^{4}$, iMIIC reaches 88% (78%) in precision (F-score), against about 60% (45%) for conservative PC, 52% (50%) for causalMGM and 22% (28%) for MXM. Finally, iMIIC reaches 86% (81%) for $N=10^{5}$, which is beyond the sample size attainable by other methods.

## Supplemental information

Document S1. Figures S1–S9

Table S1. Assessment statistics of “Putative” and “Genuine” causal edges inferred by iMIIC on SEER breast cancer 2010–2016 dataset including 396,179 patients, related to Figure 3

Table S2. All marginally dependent pairs including Death Specific Of Breast Cancer, related to Figure 3

Data S1. SEER Breast Cancer data preprocessing script, related to Figure 3

Data S2. Benchmark data generation codes and synthetic SEER-like datasets, related to Figure 1
