# Supplementary material for: Learning interpretable causal networks from very large datasets, application to 400,000 medical records of breast cancer patients
Source: iScience. 2024 Apr 16;27(5):109736. doi: 10.1016/j.isci.2024.109736 (PMC11070693; doi:10.1016/j.isci.2024.109736)
Supplement: Document S1. Figures S1–S9 [file mmc1.pdf]

iScience, Volume *27*

Supplemental information

Learning interpretable causal networks from very large datasets, application to 400,000 medical records of breast cancer patients

Marcel da Câmara Ribeiro-Dantas, Honghao Li, Vincent Cabeli, Louise Dupuis, Franck Simon, Liza Hettal, Anne-Sophie Hamy, and Hervé Isambert

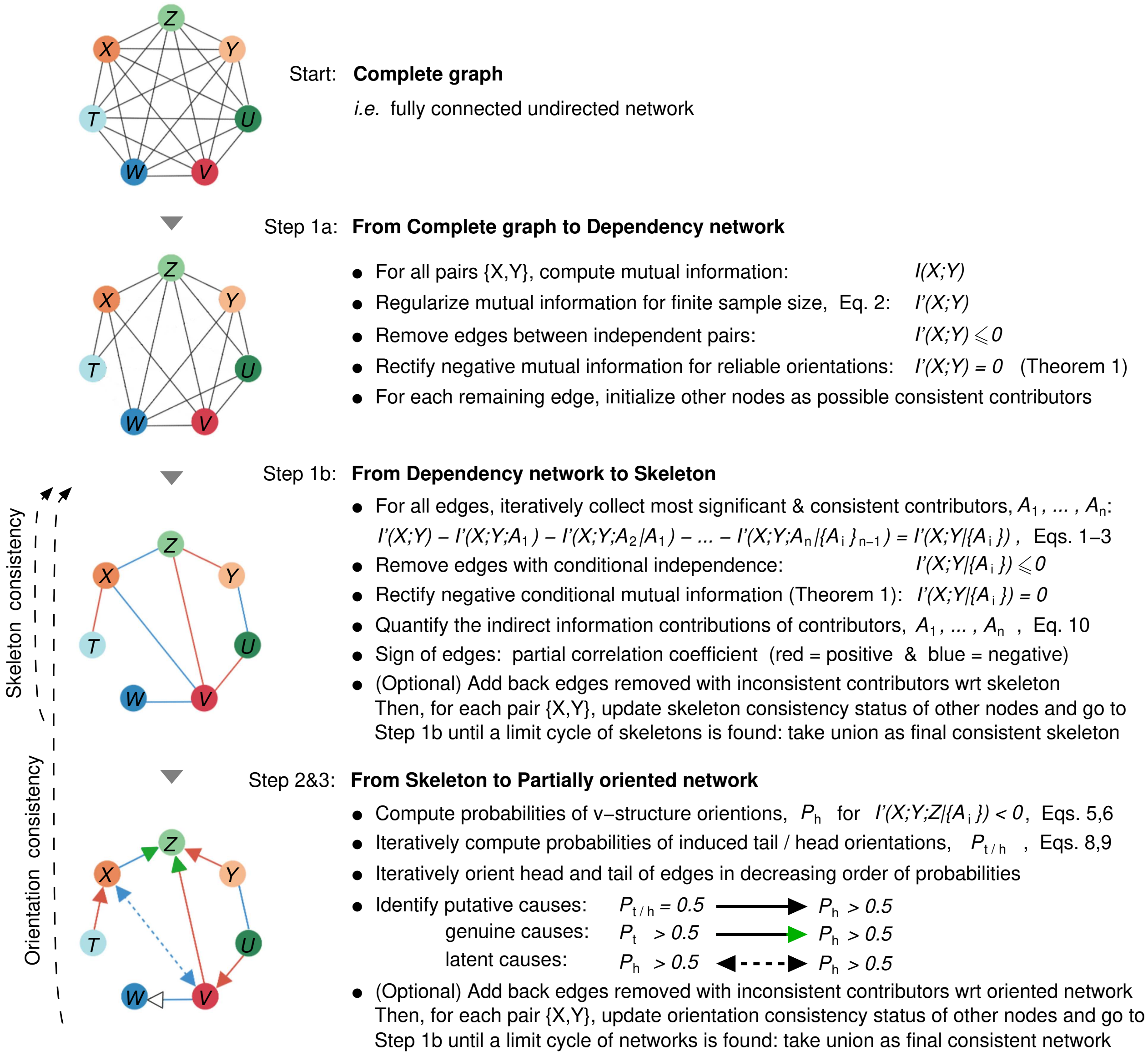

Figure S1: **iMIIC's workflow. Related to Figure 1.** Compared to the classical constraint-based algorithm scheme outlined in Figure 1b, iMIIC's workflow splits Step 1 into Step 1a and Step 1b in order to first identify the Dependency network (where all independent variables have been disconnected, Step 1a) before obtaining the skeleton (where all conditionally independent variables have also been disconnected, Step 1b). This enables to iterate either Step 1b to obtain a consistent skeleton with respect to indirect paths through the identified contributors or to iterate Step 1b and Step2&3 to obtain an orientation consistent network. By contrast, Step 2 (v-structure orientations) and Step 3 (propagation of orientations) from classical constraint-based algorithms (Figure 1b) are merged in order to orient, on the same footing, v-structures and induced head / tail orientations ranked by decreasing probabilities. The rectification of negative (conditional) mutual information, required by Theorem 1, is shown to improve the reliability of head and tail orientations in Step 2&3, see Method Details. Finally, iMIIC also distinguishes genuine causal relations from putative and latent causal effects in Step 2&3.

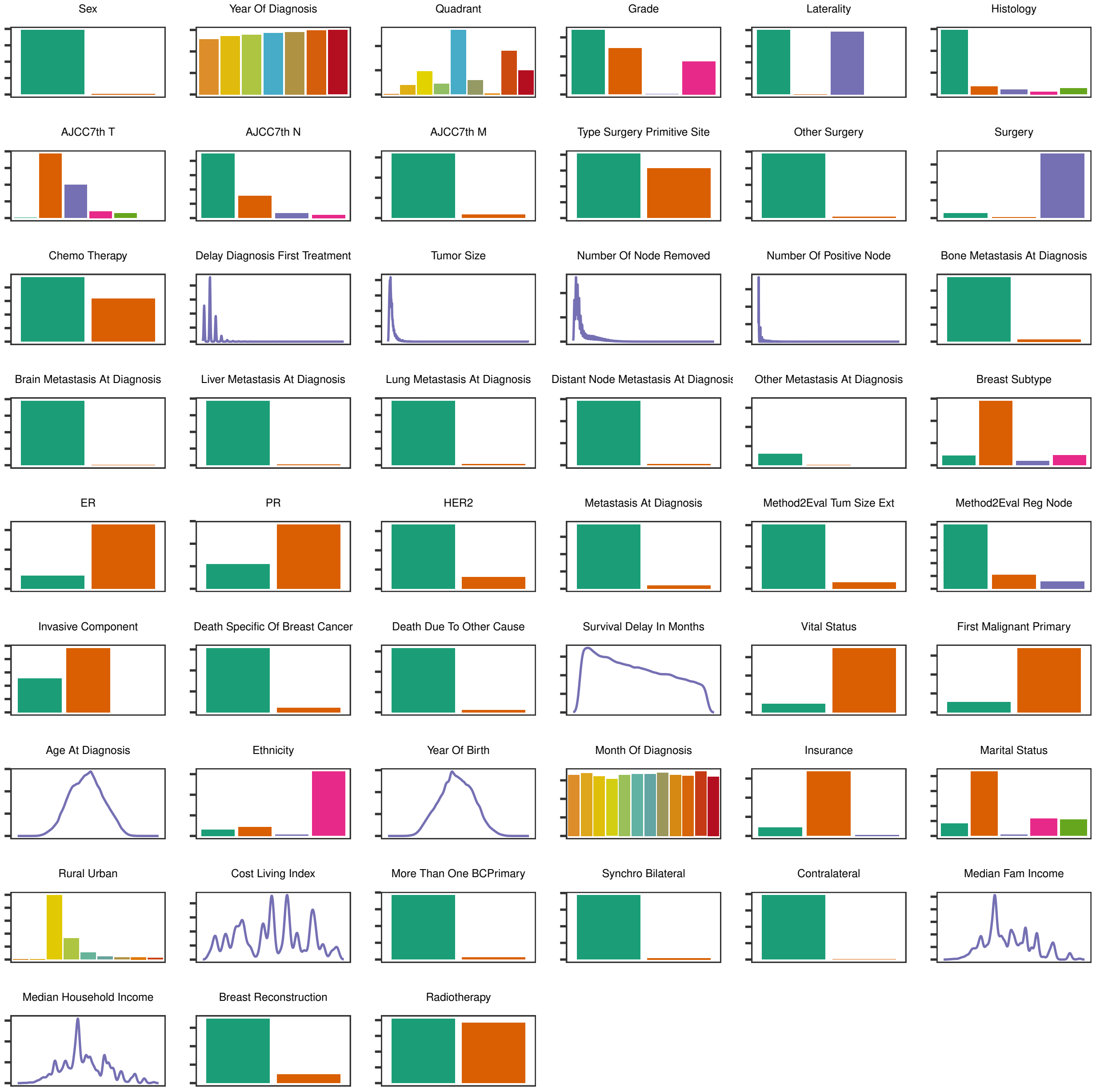

Figure S2: **Distributions of the 51 SEER variables selected for breast cancer. Related to Figure 1.** The preprocessing of SEER breast cancer data for the period of 2010-2016 is described in Method Details and leads to a selection of 51 relevant variables for breast cancer data.

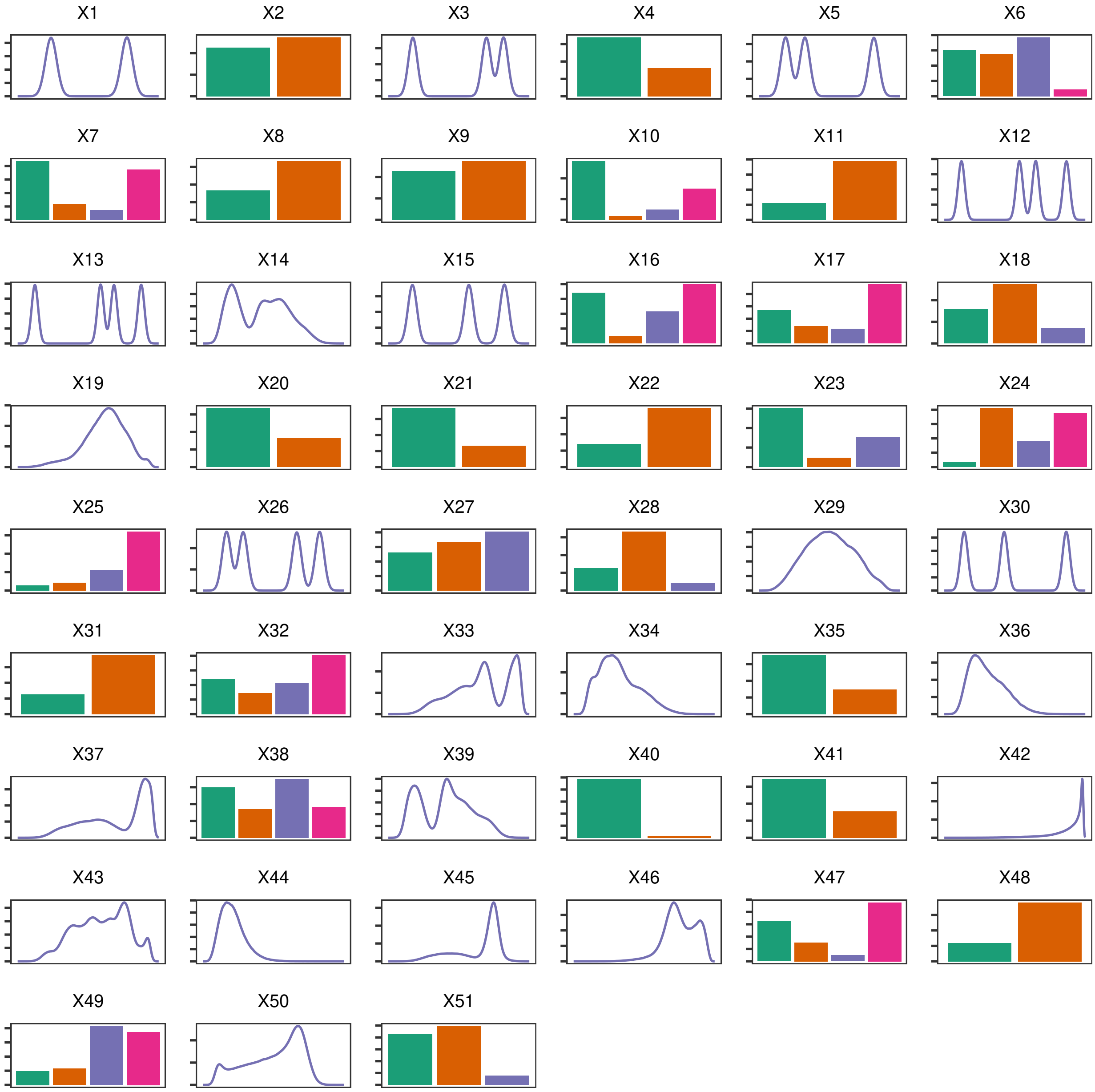

Figure S3: **Example of simulated SEER-like dataset. Related to Figure 1.** Example of marginal distributions of simulated SEER-like datasets (including about 60% of discrete variables here) obtained using mixed-type structural equation models (SEMs), see SEER-like data generation in the Quantification and Statistical Analysis section of Methods.

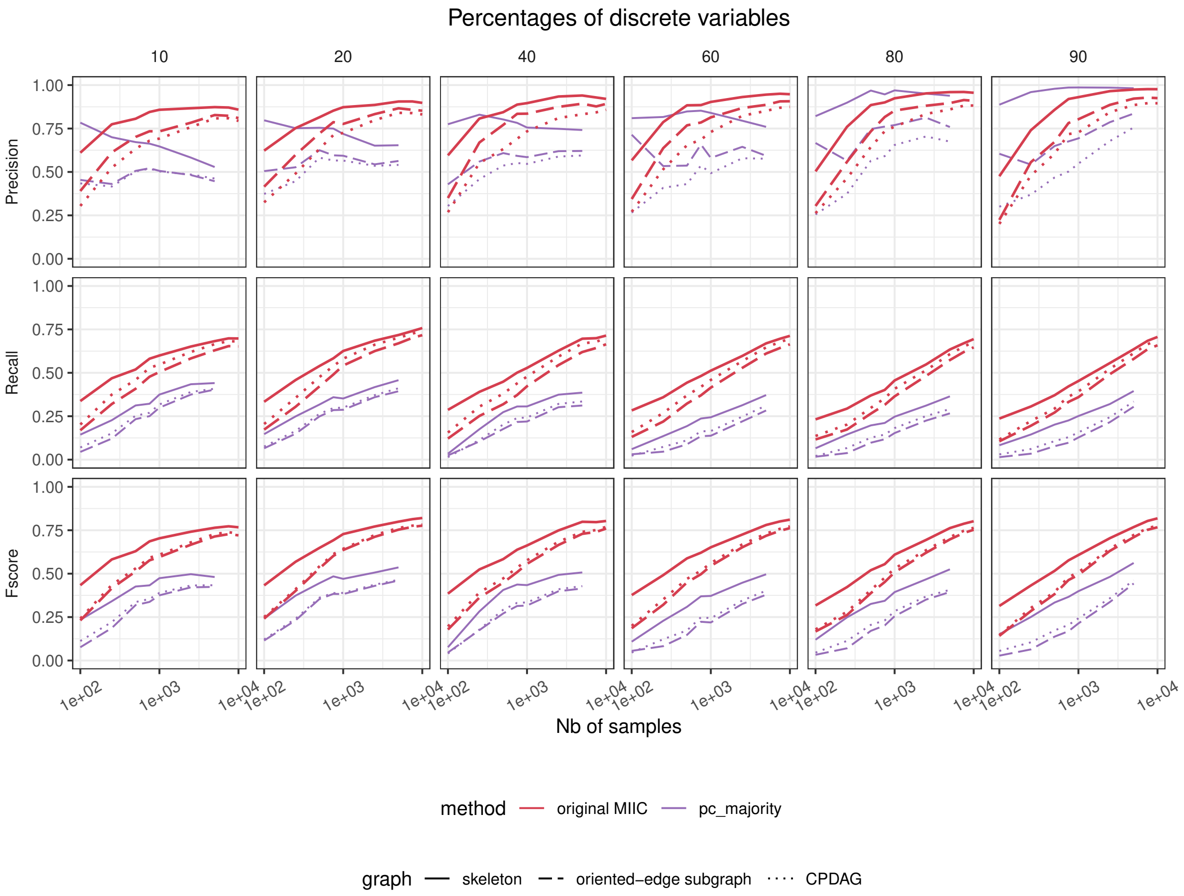

Figure S4: **Original MIIC *versus* PC on SEER-like benchmarks. Related to Figure 1.** See parameter settings and causal discovery scores in the Quantification and Statistical Analysis section of Methods. Oriented-edge subgraph scores (dashed lines) are restricted to the subgraphs containing only oriented edges in the theoretical CPDAG *versus* the inferred graph. These oriented-edge scores are designed to specifically assess the method performance on causal discovery, that is, on the oriented edges which can in principle be learnt from observational data *versus* those effectively predicted by the causal structure learning method.

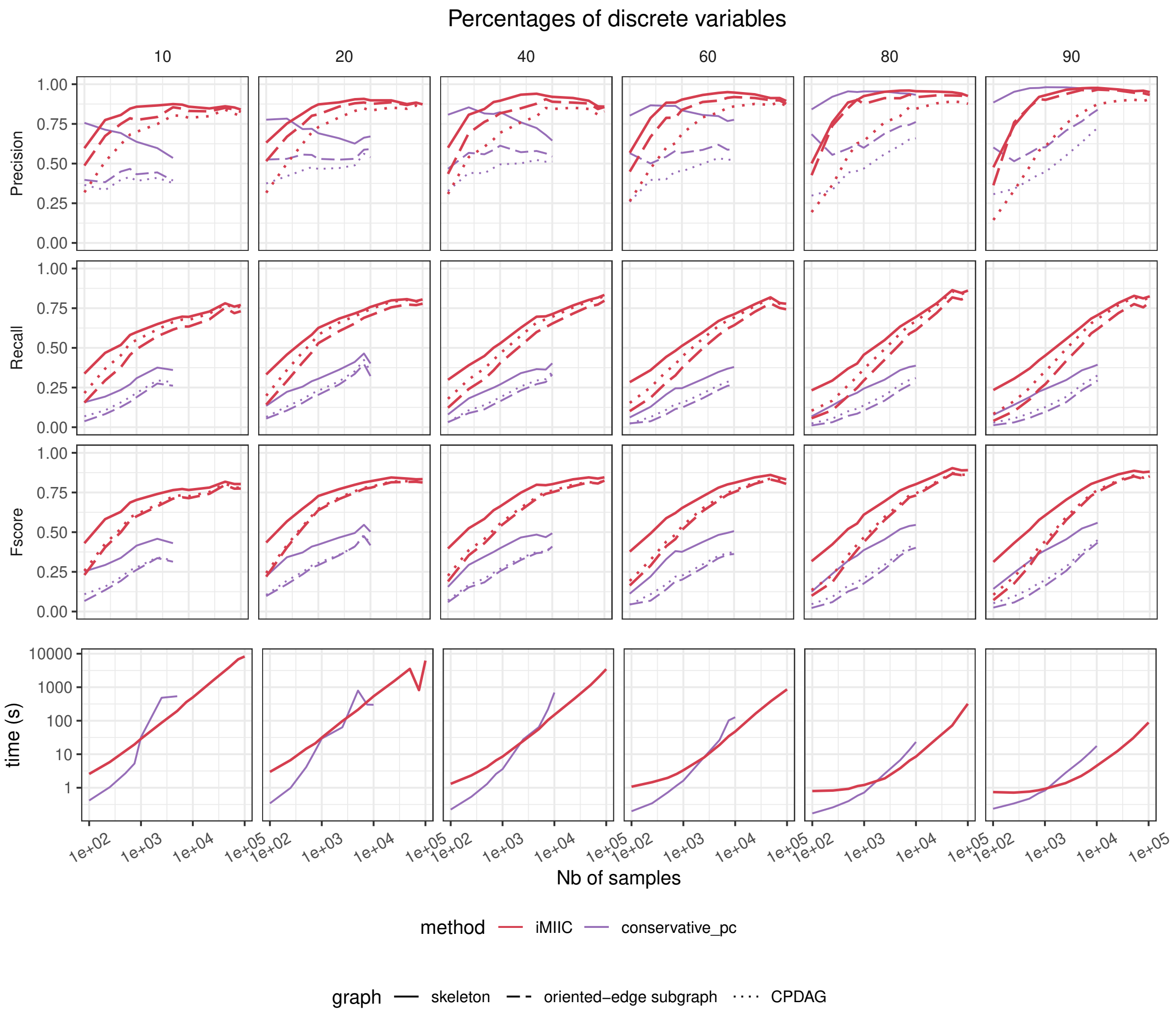

Figure S5: **iMIIC *versus* PC on SEER-like benchmarks. Related to Figure 1.** See parameter settings and causal discovery scores in the Quantification and Statistical Analysis section of Methods. Oriented-edge subgraph scores (dashed lines) are restricted to the subgraphs containing only oriented edges in the theoretical CPDAG *versus* the inferred graph. These oriented-edge scores are designed to specifically assess the method performance on causal discovery, that is, on the oriented edges which can in principle be learnt from observational data *versus* those effectively predicted by the causal structure learning method.

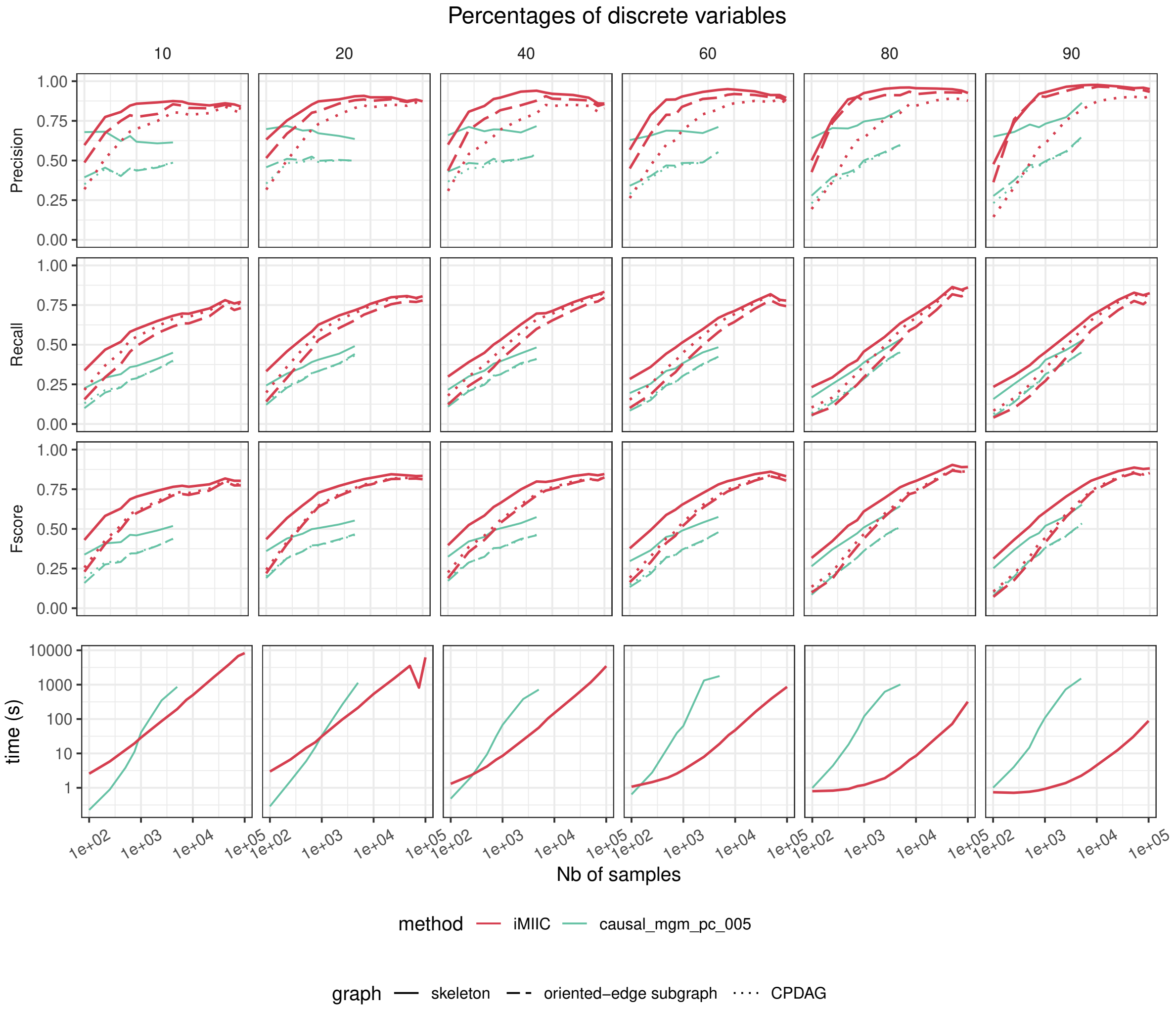

Figure S6: **iMIIC *versus* causalMGM on SEER-like benchmarks. Related to Figure 1.** See parameter settings and causal discovery scores in the Quantification and Statistical Analysis section of Methods. Oriented-edge subgraph scores (dashed lines) are restricted to the subgraphs containing only oriented edges in the theoretical CPDAG *versus* the inferred graph. These oriented-edge scores are designed to specifically assess the method performance on causal discovery, that is, on the oriented edges which can in principle be learnt from observational data *versus* those effectively predicted by the causal structure learning method.

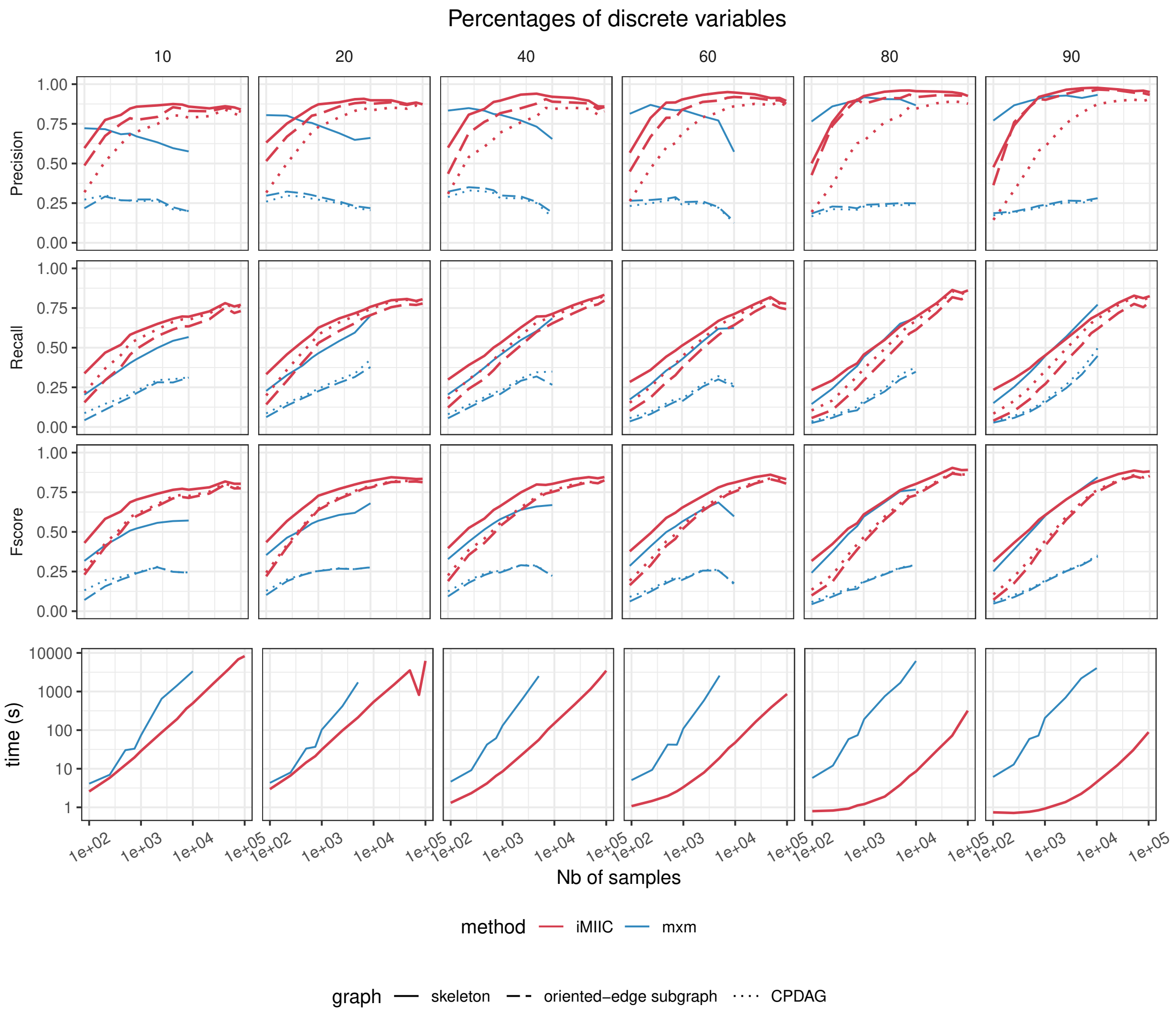

Figure S7: **iMIIC *versus* MXM on SEER-like benchmarks. Related to Figure 1.** See parameter settings and causal discovery scores in the Quantification and Statistical Analysis section of Methods. Oriented-edge subgraph scores (dashed lines) are restricted to the subgraphs containing only oriented edges in the theoretical CPDAG *versus* the inferred graph. These oriented-edge scores are designed to specifically assess the method performance on causal discovery, that is, on the oriented edges which can in principle be learnt from observational data *versus* those effectively predicted by the causal structure learning method.

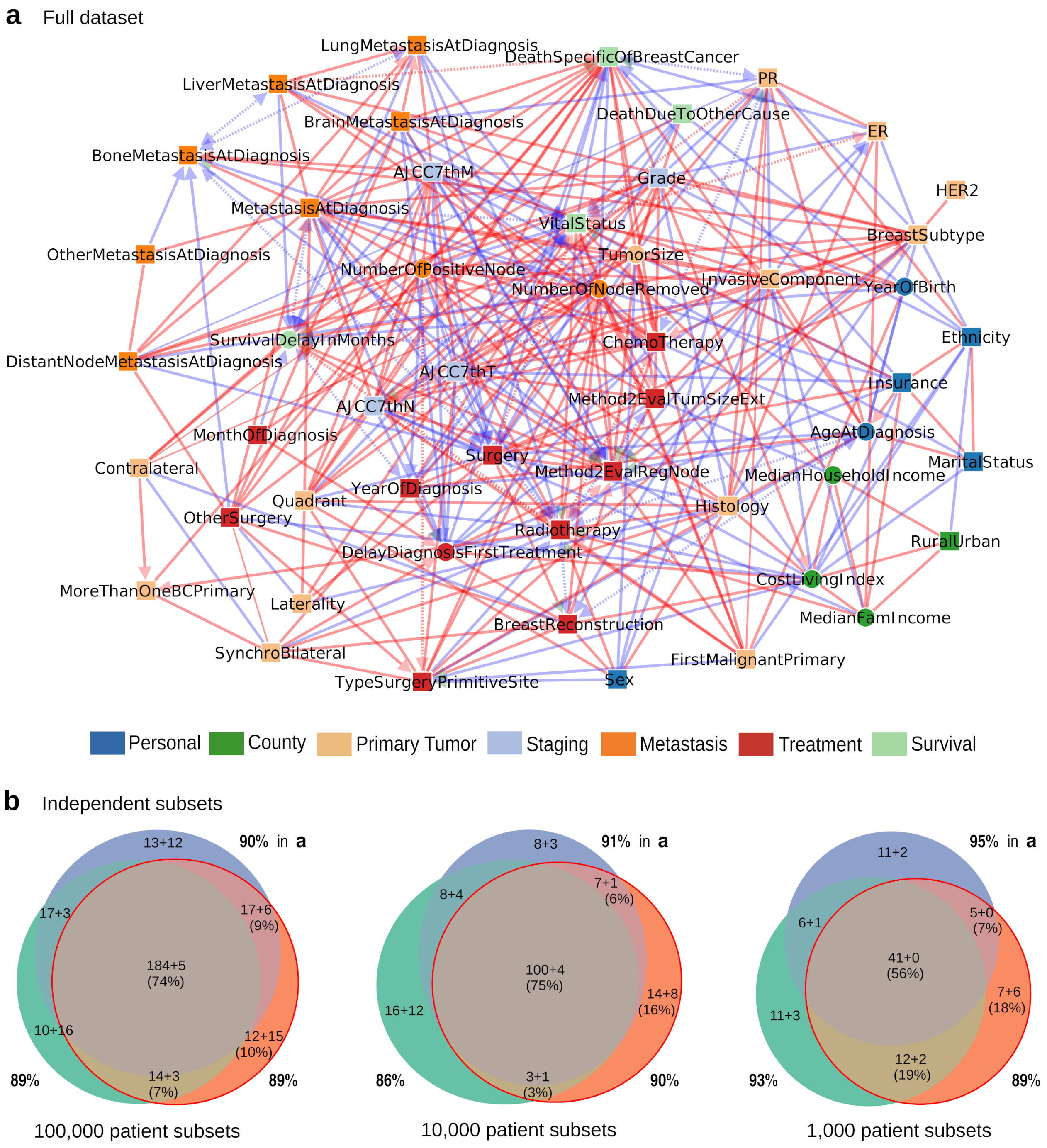

Figure S8: **SEER breast cancer orientation consistent networks inferred by iMIIC. Related to Figure 3. (a)** The 51 node network inferred by iMIIC from SEER dataset containing 396,179 breast cancer patients diagnosed between 2010 and 2016. This orientation consistent network contains 340 edges and includes 2 contextual variables, Sex and Year of birth. Red (resp. blue) edges indicate correlated (resp. anticorrelated) variables. 'Genuine' causal edges are shown with green arrowheads and 'putative' causal relations with red or blue arrowheads, while bidirected dashed edges correspond to the effect of unobserved latent variables (Figure 2). See Table S1 for a list and causal nature of each edges predicted by iMIIC. **(b)** Comparisons of networks inferred from three independent sub-samplings of the same size of 100,000, 10,000 or 1,000 patient subsets (from left to right). Number of shared edges (regardless of orientations) in the Euler diagrams are given as a sum $a + b$ where a (resp. b) corresponds to the number of edges included in (resp. absent from) the full dataset network in (a). Percentages in brackets refer to the subset network with the median total number of edges (red circle). The fractions of edges also found in the full network in (a) are indicated around the Euler diagram for each independent subset. These fractions of shared edges are high (*i.e.* 86-95%) for all independent subset sizes, demonstrating that iMIIC reconstructs essentially subnetworks of the full network for all independent subset sizes, with a decreasing overlap between smaller subnetworks learnt from smaller independent subsets, see main text.

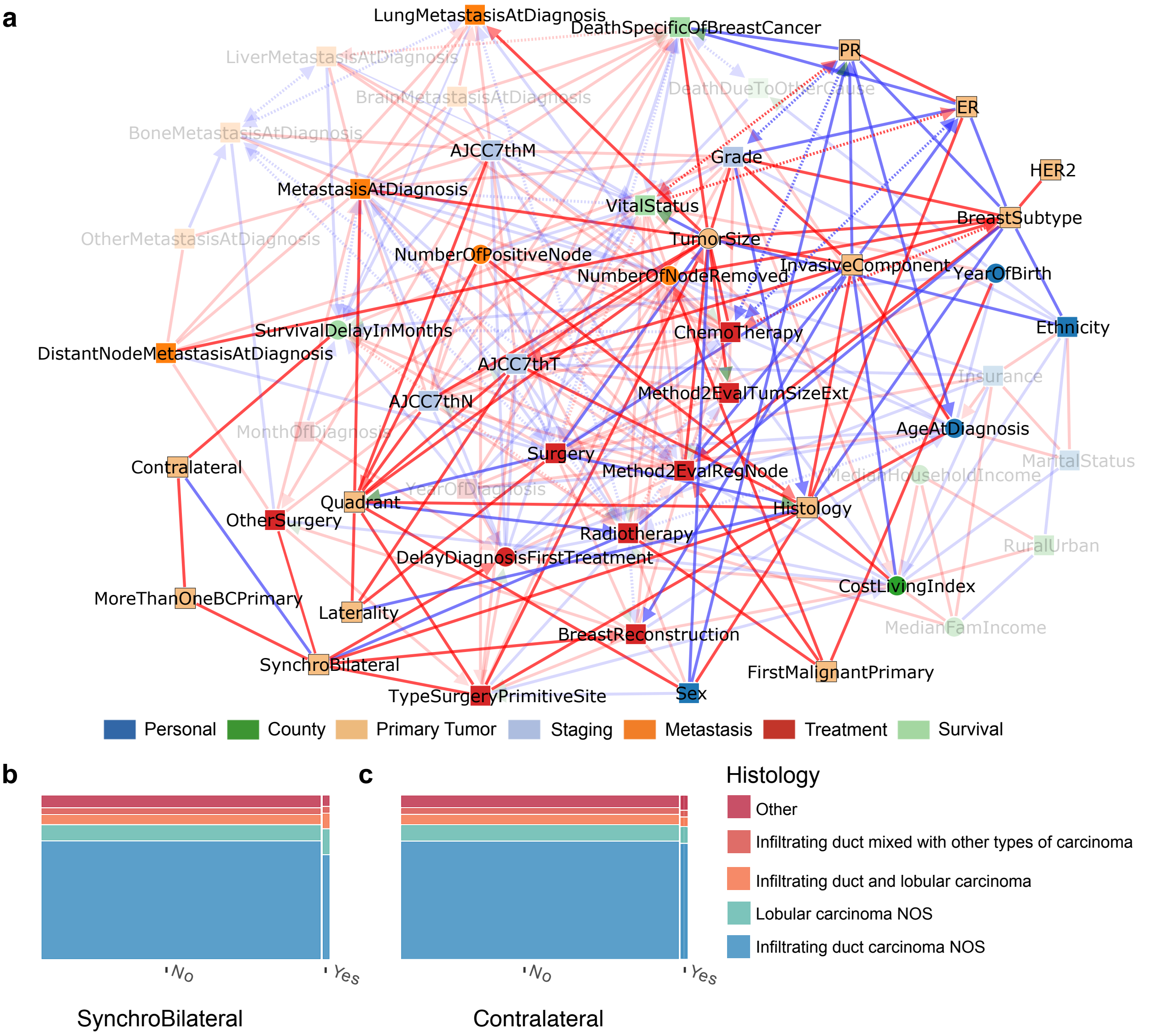

Figure S9: **Primary Tumor subnetwork inferred by iMIIC from SEER breast cancer dataset. Related to Figure 3.** **(a)** Subnetwork highlighting direct relations with primary tumor variables (Contralateral, MoreThanOneBCPrimary, SynchroBilateral, Laterality, Quadrant, Histology, FirstMalignantPrimary, TumorSize, InvasiveComponent, PR, ER, HER2, and BreastSubtype). **(b)** Joint distribution of Histology and Synchro Bilateral tumor. **(c)** Joint distribution of Histology and Contralateral tumor, see main text.
