# Supplementary figures and images for: Learning interpretable causal networks from very large datasets, application to 400,000 medical records of breast cancer patients
Source: iScience. 2024 Apr 16;27(5):109736. doi: 10.1016/j.isci.2024.109736 (PMC11070693; doi:10.1016/j.isci.2024.109736)

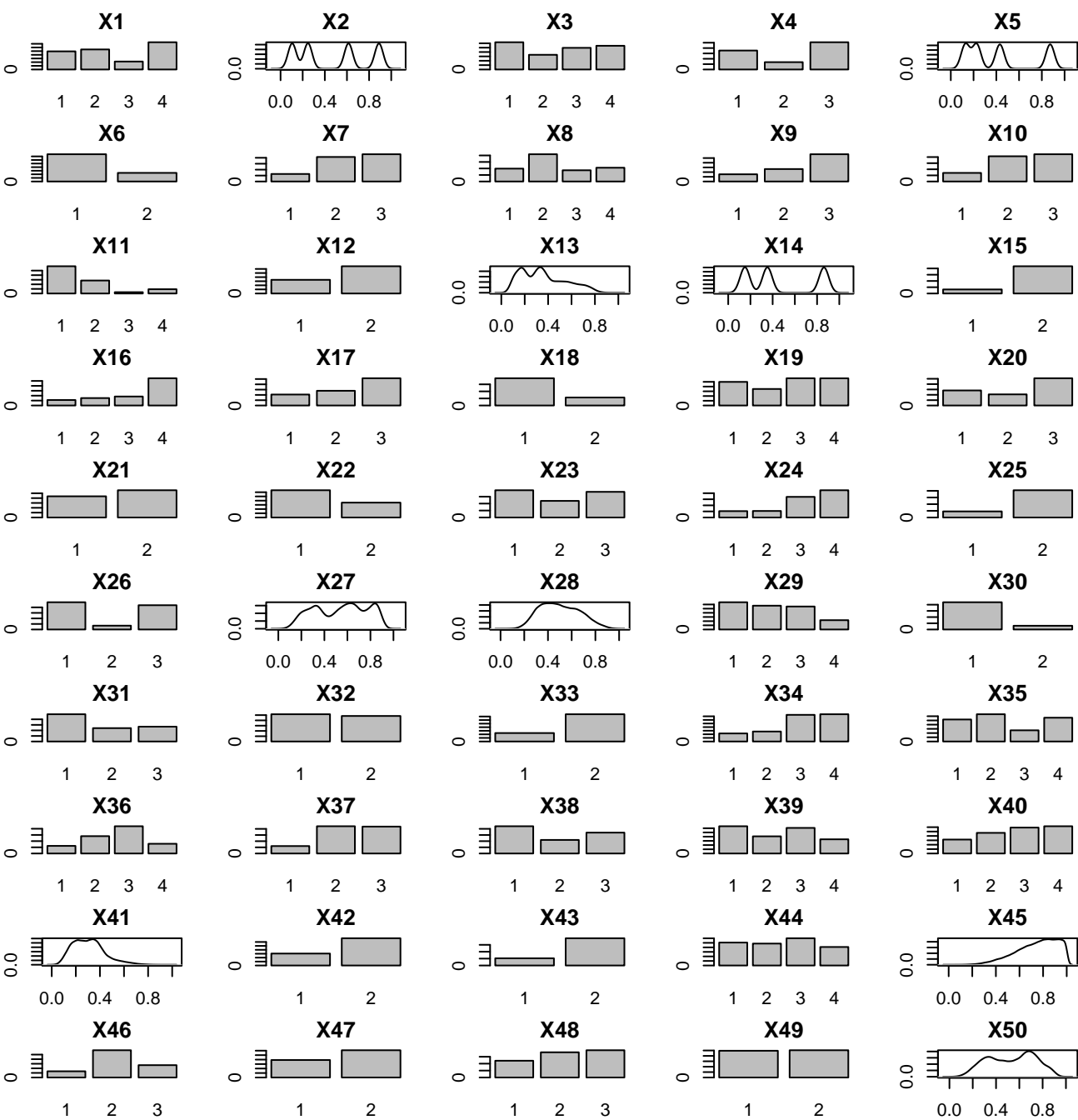

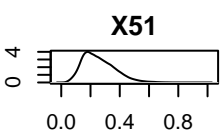

Supplement: Data S2. Benchmark data generation codes and synthetic SEER-like datasets, related to Figure 1 [file mmc5.zip › benchmark_example/synthetic_network_example/synthetic_data_distributions_N-1-51.pdf]

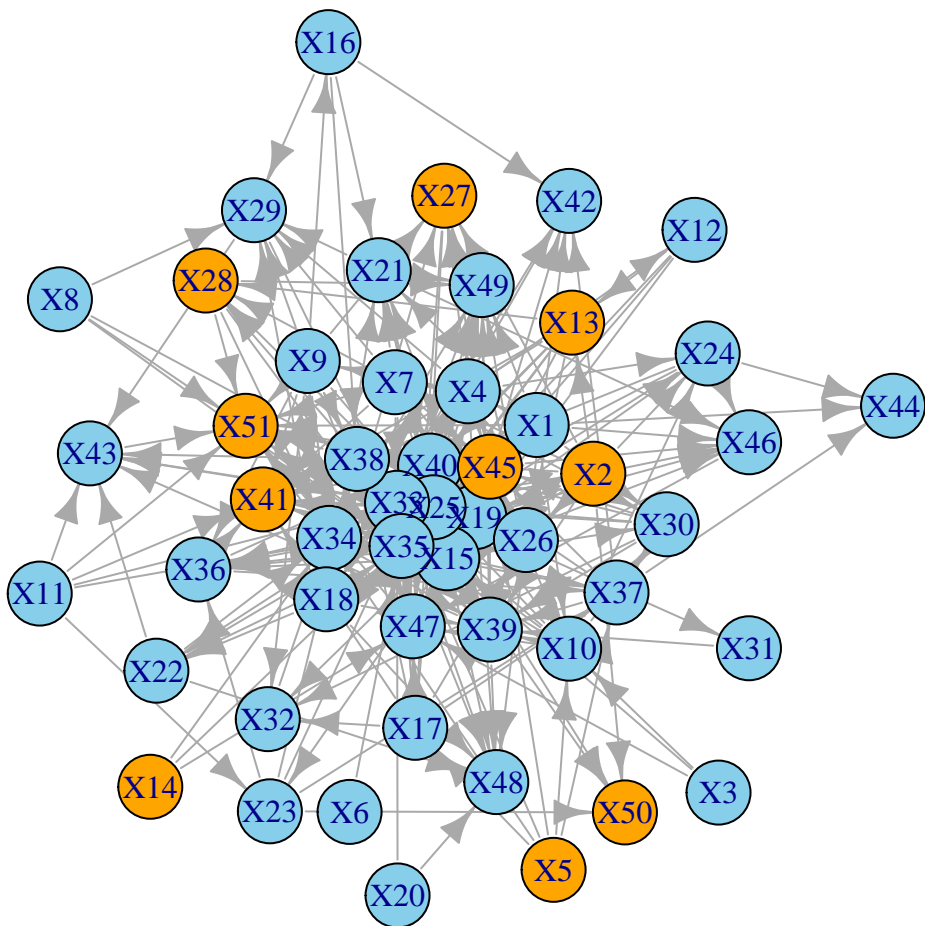

Supplement: Data S2. Benchmark data generation codes and synthetic SEER-like datasets, related to Figure 1 [file mmc5.zip › benchmark_example/synthetic_network_example/synthetic_network_graph.pdf]
